# Critical signaling molecules in the temporomandibular joint osteoarthritis under different magnitudes of mechanical stimulation

**DOI:** 10.3389/fphar.2024.1419494

**Published:** 2024-07-11

**Authors:** Yuqi Liu, Fangwen Jia, Kangya Li, Chao Liang, Xiao Lin, Wei Geng, Yanxi Li

**Affiliations:** Department of Dental Implant Center, Beijing Stomatological Hospital, School of Stomatology, Capital Medical University, Beijing, China

**Keywords:** temporomandibular joint, mechanical stimulation, condylar cartilage, chondrocyte, osteoarthritis

## Abstract

The mechanical stress environment in the temporomandibular joint (TMJ) is constantly changing due to daily mandibular movements. Therefore, TMJ tissues, such as condylar cartilage, the synovial membrane and discs, are influenced by different magnitudes of mechanical stimulation. Moderate mechanical stimulation is beneficial for maintaining homeostasis, whereas abnormal mechanical stimulation leads to degeneration and ultimately contributes to the development of temporomandibular joint osteoarthritis (TMJOA), which involves changes in critical signaling molecules. Under abnormal mechanical stimulation, compensatory molecules may prevent degenerative changes while decompensatory molecules aggravate. In this review, we summarize the critical signaling molecules that are stimulated by moderate or abnormal mechanical loading in TMJ tissues, mainly in condylar cartilage. Furthermore, we classify abnormal mechanical stimulation-induced molecules into compensatory or decompensatory molecules. Our aim is to understand the pathophysiological mechanism of TMJ dysfunction more deeply in the ever-changing mechanical environment, and then provide new ideas for discovering effective diagnostic and therapeutic targets in TMJOA.

## 1 Introduction

Frequent mandibular movements due to daily biting, chewing and speaking lead to alterations in condylar position within the temporomandibular joint (TMJ), thereby changing the mechanical loading on the condyle (Feng et al., 2021). Moderate mechanical loading is essential for maintaining the normal structure and function of the TMJ (Robinson et al., 2019). Adequate loading is required to prevent atrophy of the mandibular condylar fibrocartilage (Pirttiniemi et al., 2004). However, abnormal mechanical loading caused by poor prosthesis, occlusal interference, trauma and bruxism contributes to degenerative changes in TMJ tissues, eventually resulting in temporomandibular joint osteoarthritis (TMJOA) (Teramoto et al., 2003; Liu et al., 2021a).

Condylar cartilage is a critical component of the TMJ and is mechanosensitive. Condylar chondrocytes are responsible for regulating the balance between extracellular matrix (ECM) synthesis and degradation (Li et al., 2021). Moderate mechanical stimulation can promote ECM synthesis (Rabie et al., 2003a; Chen et al., 2007). In contrast, abnormal mechanical stimulation enhances catabolic effects, thereby disturbing the homeostasis of the cartilage matrix followed by cartilage degradation (Li et al., 2014), which involves changes in critical signaling molecules. Under abnormal mechanical stimulation, some molecules play a compensatory role by impeding degenerative changes, whereas other molecules play a decompensatory role by accelerating cartilage degeneration.

Most current reviews have focused on the pathological mechanism of TMJOA occurrence and development (Wang et al., 2015; Liu et al., 2021a; Li et al., 2021). One recent review summarized the molecular signaling pathways involved in TMJOA, but the authors paid more attention to the molecules with decompensatory effects under abnormal stimulation that promote TMJOA progression (Lu et al., 2022). Additionally, a systematic review discussed the different effects of different types and magnitudes of mechanical loading on the TMJ but did not elucidate the detailed functions of critical signaling molecules (Betti et al., 2018). To our knowledge, there are no reviews discussing changes in signaling molecules with compensatory or decompensatory effects in the TMJ under moderate or abnormal mechanical stimulation.

In this review, we mainly describe the signaling molecules in condylar chondrocytes and cartilage. We first introduce elements of mechanotransduction in chondrocytes, and critical signaling molecules under moderate and abnormal mechanical stimulation are covered separately. In a section on abnormal mechanical stimulation, we divide signaling pathways into two categories according to whether they have compensatory or decompensatory effects. In addition, we investigate molecular signaling changes in the subchondral bone, synovial membrane and disc. Our purpose is to provide a better understanding of the pathophysiologic mechanism of TMJ dysfunction under different magnitudes of mechanical stimulation.

## 2 Search strategy

A literature search was performed mainly in Web of Science up to May 2024. Keywords with different combinations of “TMJOA”, “TMJ”, “temporomandibular joint osteoarthritis”, “temporomandibular joint”, “mechanical”, “mechanosensitive”, “mechanical stimulation”, “mechanical loading”, “mechanical stress”, “chondrocyte”, “cartilage”, “synovial membrane”, “disc” were used. Basic researches related to mechanical stimulation were included. Clinical researches or basic researches not related to mechanical stimulation were excluded. In addition, reference lists of potential related original articles and reviews were screened manually to identify any researches that could have been overlooked.

## 3 Elements in mechanotransduction

**FIGURE 1:**
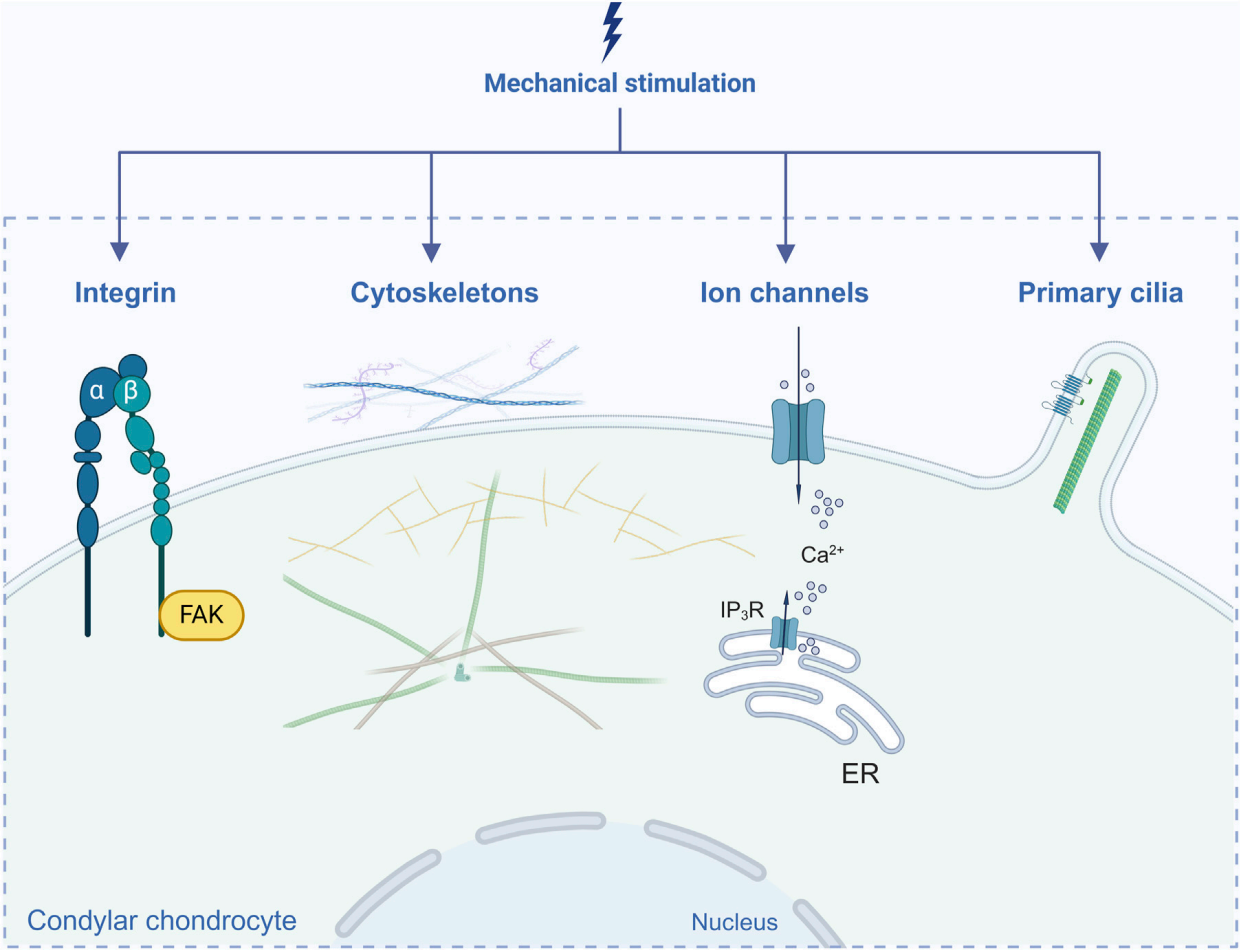
Elements in mechanotransduction reported in condylar chondrocytes. Created with BioRender.com.

For Under mechanical loading, cells respond to physical stimuli and convert into biochemical signals, which induce a series of cellular responses followed by changes in cell phenotype as well as the structure and composition of the ECM. This process is called mechanotransduction and is dependent on mechanosensitive elements, mainly integrins, the cytoskeleton, ion channels and primary cilia (Hodgkinson et al., 2022; Wang et al., 2023). In this section, we focus on reported elements in condylar chondrocytes (Figure 1).

### 3.1 Integrins

Integrins, a group of mechanosensitive cell-surface receptors consisting of α and β subunits, mediate extracellular-intracellular signaling transduction and subsequently activate several intracellular cascades, which are essential for the maintenance of homeostasis and regulation of cellular biological functions (Humphries, 2000; Kechagia et al., 2019). αVβ1, αVβ3, αVβ5, α1β1, α3β1, α5β1 and α10β1 are the major integrins expressed in chondrocytes (Wang et al., 2023). High expression levels of α5β1 and αVβ3 in the hypertrophic layer of condylar cartilage have been reported (Yanoshita et al., 2020). Both the αv and α5 integrin subunits are receptors for fibronectin. It was found that a mandibular propulsion appliance enhances expression of the fibronectin, αv and α5 integrin subunits in the proliferative compartment of rat condylar cartilage, suggesting that the mechanical force is transduced into the proliferative signal (Marques et al., 2008). One study showed that under short-term (90 kPa/1 h) or long-term (90 kPa/6 h) pressure, the α5 and β1 subunits of condylar chondrocytes presented different variations: the α5 subunit was downregulated with time but the β1 subunit upregulated (Zhang et al., 2008). In addition, expression of the β2 subunit in condylar cartilage was altered according to TMJOA-like progression in a rat model of lateral mandibular shift (Zou et al., 2022). Therefore, different integrin subunits may play different roles.

Focal adhesion kinase (FAK) is one of the main adaptor molecules involved in intracellular integrin signaling. Liu et al. demonstrated that compared with those on the contralateral side, mechanical stress (induced by a rat model of mandibular lateral shift on the ipsilateral side) enhances expression of integrin α5β1, FAK and integrin-linked kinase (ILK) in condylar cartilage at the early stage (Liu et al., 2008). Another study showed that expression of integrin α2, α5 and β1 in primary condylar chondrocytes was enhanced in a dose-dependent manner under different magnitudes (50–250 kPa) of hydrostatic compressive forces (HCFs), followed by increased phosphorylation levels of FAK, ERK1/2 (extracellular signal–regulated kinase 1/2) and PI3K (phosphatidylinositol-3-kinase). The authors confirmed that HCFs reduced apoptosis and enhanced the viability of condylar chondrocytes via the integrin-FAK-ERK/PI3K pathway (Ma et al., 2016). However, under excessive mechanical loading, phosphorylation of FAK (pFAK) was related to signaling dysfunction during TMJOA. Moreover, inhibiting pFAK moderately slowed OA progression (Reed et al., 2021).

### 3.2 The cytoskeleton

The cytoskeleton is capable of sensing mechanical stimuli to induce rapid remodeling and functional changes. This process involves intact microtubules (tubulins), microfilaments (actins) and intermediate filaments (vimentin) (Wang et al., 1993). For condylar chondrocytes, Zhang et al. reported that the cytoskeleton exhibited a tighter arrangement under proper pressure of 90 kPa for 60 min. However, as time extended to 360 min, the arrangement became loose with a decrease in intracellular communication function (Zhang et al., 2006). In another study, 2,000 µstrain loading did not induce significant changes in the cytoskeleton. However, under 4,000 µstrain, partial actin filaments accumulated immediately accompanied by cytoskeletal rearrangement, and the cell cycle was inhibited (Li et al., 2010). In addition, with the contractibility of actin filaments, proteins associated with the cytoskeleton can be activated. A cyclical uniaxial compressive stress of 2,000 µstrain for 2 h has been proven to activate myosin light chain II (MLC-II) and subsequently promote condylar chondrocyte differentiation (Liu et al., 2016). Vimentin is more sensitive than other cytoskeletal proteins because of its faster response. In a mechanical stress loading rat model, there was a significant decrease in the thickness of condylar cartilage as well as in vimentin expression at 7 days, which suggested that downregulation of vimentin probably results in destructive morphological changes in cartilage (Li et al., 2010).

### 3.3 Ion channels

Mechanical stimuli can lead to fluctuations in ion signaling in chondrocytes though activation of ion channels (Agarwal et al., 2021). Calcium is one of the most ubiquitous second messengers. Intracellular Ca^2+^ and calcium channels play essential roles in mechanotransduction signaling (Jiang et al., 2021). Transient receptor potential cation channel subfamily V member 4 (TRPV4) and Piezo1/2 are typical Ca^2+^ ion channels. Under moderate mechanical stress, activation of TRPV4 has been confirmed to mediate anabolic responses (O’Conor et al., 2014). However, under excessive mechanical stress, high TRPV4 expression and enhanced Ca^2+^ influx can induce chondrocyte apoptosis (Xu et al., 2019) and promote degenerative changes in the TMJ disc (Cui et al., 2023). Piezo1 and Piezo2 are also Ca^2+^-permeable channels in chondrocytes that are activated under abnormal strain (Du G. et al., 2020). On the basis of the findings of Servin-Vences et al., only Piezo1, and not Piezo2 or TRPV4, responds to stretch-activated currents (Servin-Vences et al., 2017). Zhang et al. confirmed that condylar chondrocytes respond to cyclic tensile strain (CTS) with 20% elongation of 0.1 Hz via Piezo1 followed by downregulation of sex-determining region Y-box 9 (SOX9) and COL2A1 (Zhang et al., 2022a). A recent study showed that in a unilateral anterior crossbite (UAC) rat model, Piezo1 was overexpressed in condylar cartilage, promoting progression of TMJOA through the Yes-associated protein (YAP)-matrix metalloproteinase 13 (MMP13)/a disintegrin and metalloproteinase with thrombospondin motif 5 (ADAMTS5) signaling pathway (Feng et al., 2023). On the one hand, TRPV4 and Piezo1 can act independently. Du et al. reported that moderate stretching-induced Ca^2+^ flux was significantly inhibited after knockout of TRPV4 but that the cell response to excessive stretching was not affected (Du G. et al., 2020). On the other hand, TRPV4 and Piezo1 have been proven to communicate with each other, and their crosstalk may be impaired in a state of inflammation (Steinecker-Frohnwieser et al., 2023).

In addition to TRPV4 and Piezo1, other kinds of Ca^2+^-permeable channels have been reported. Under moderate pressure (90 kPa) for 60 min, the inositol triphosphate (IP_3_) channel on the endoplasmic reticulum (ER) becomes activated, resulting in a higher intracellular Ca^2+^ concentration in condylar chondrocytes (Zhang et al., 2006). Inhibiting inositol trisphosphate receptor (IP_3_R) channels with 2-aminoethoxydiphenyl borate (2APB) or inhibiting ryanodine receptor (ROR) channels with ryanodine (Rya) can block [Ca^2+^]i accumulation, attenuating the condylar cartilage degeneration induced by compressive mechanical force (Zhu et al., 2016). Wei et al. reported that TRPV5 was upregulated in a rat model of mechanical stress-induced OA, which enhanced Ca^2+^ influx and subsequently promoted chondrocyte apoptosis via the calmodulin-dependent protein kinase II (CaMKII)-mitogen-activated protein kinase (MAPK) and Akt/mammalian Target of rapamycin (mTOR) pathways (Wei et al., 2018).

In brief, different ion channels in chondrocytes may respond differently to mechanical stimulation. However, the interactions among them are still not fully understood and deserve further exploration.

### 3.4 Primary cilia

The primary cilium is a nonmotile cytoskeletal organelle containing microtubules that protrude from the cell surface into the pericellular matrix (Tao et al., 2020). Primary cilia play a critical role in mechanotransduction in chondrocytes (Hodgkinson et al., 2022). Mechanobiological signal transduction is impaired when the primary cilium structure is disrupted by chloral hydrate (Shao et al., 2012). In response to mechanical stress, the primary cilium is able to bend or change in length, and bending of primary cilia promotes secretion of the ECM (Jensen et al., 2004). However, high levels of mechanical loading can induce cilia disassembly, which results in chondroprotective effects by preventing hedgehog signaling and ADAMTS-5 expression (Thompson et al., 2014). Further exploration is needed to determine how the length of cilia changes in response to mechanical stimulation.

In fact, primary cilia serve as compartments containing high densities of mechanosensory elements, such as ion channels, connexins and intraflagellar transport (IFT) proteins (Ruhlen and Marberry, 2014; Hodgkinson et al., 2022). Connexin 43 is a mechanosensitive hemichannel that mediates small molecule exchange (Knight et al., 2009). In a UAC rat model, Connexin 43 mediated exchange of prostaglandin E2 (PGE2) in condylar cartilage, which contributed to catabolic changes (Zhang et al., 2014). IFT is the main biological activity of primary cilia and IFT88 is one of the core proteins essential for cilium formation and maintenance of cartilage homeostasis (Ding et al., 2017; Zhao et al., 2020; Coveney et al., 2022). The articular cartilage of IFT88 knockout mice exhibited OA-like features (Chang et al., 2012). A recent study indicated that IFT88 determines the integrity of cilia and regulates the level of Piezo1. The authors confirmed the synergistic interaction between IFT88 and Piezo1 in regulating condylar chondrocyte differentiation under cyclic tensile strain (Zhang et al., 2022a). In addition, polycystin 2 was found to be an essential subunit for the ion channel located within the primary cilium; this subunit mainly conducts Na^+^ and K^+^ and is enhanced by Ca^2+^ (Liu X. et al., 2018). IFT88 and polycystin 2 coordinately regulate hedgehog signaling in condylar chondrocytes after cyclic tensile strain stimulation (Wang Z. et al., 2022).

Notably, the four abovementioned elements are inseparable and interact with each other. For example, when the IP_3_R channel is blocked under mechanical pressure, the intracellular Ca^2+^ concentration decreases, disrupting cytoskeletal reorganization (Zhang et al., 2006). They can also be affected by other signaling molecules. Indeed, there is evidence suggesting that the signaling pathways mediated by traditional integrin and G proteins coregulate the function of condylar chondrocytes (Zhang et al., 2008). The detailed downstream signaling molecules involved in the initiation and development of TMJOA after mechanotransduction are discussed in the following sections.

## 4 Moderate mechanical stimulation

**FIGURE 2:**
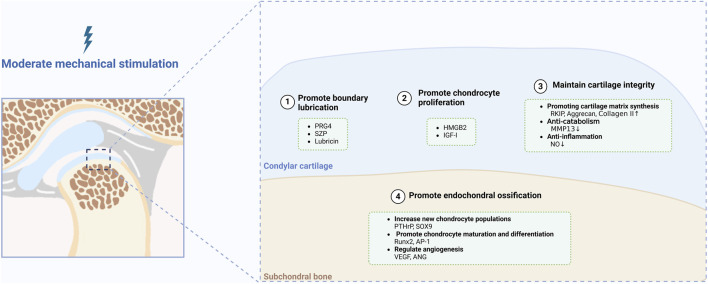
Critical signaling molecules under moderate mechanical stimulation. Created with BioRender.com.

Moderate mechanical stimulation is necessary for maintaining the normal functions and homeostasis of the condyle. Mice with incisor trimming and a soft diet demonstrated impaired condylar cartilage and subchondral bone (Chen et al., 2009). Decreased mechanical loading upon condylar cartilage results in degenerative changes via the YAP/light chain 3 (LC3)/Runt-related transcription factor 2 (RUNX2) signaling pathway (Hou et al., 2023). In this section, we focus on essential signaling molecules involved in the maintenance of homeostasis under moderate mechanical stimulation, which are mainly associated with boundary lubrication, proliferation, maintenance of integrity and endochondral ossification (Figure 2).

### 4.1 Promotion of boundary lubrication

The articular cartilage usually has a frictionless surface due to the presence of a boundary lubricating layer (Seror et al., 2015). Proteoglycan 4 (PRG4), a mucinous glycoprotein secreted by superficial zone chondrocytes and synovial fibroblasts, is considered to play an essential role in joint boundary lubrication (Schumacher et al., 1994; Flannery et al., 1999; Schmidt et al., 2004). Moreover, PRG4 exerts other biological effects, such as improving subchondral bone remodeling and preventing chondrocyte apoptosis as well as protein deposition on the cartilage surface (Rhee et al., 2005; Jay and Waller, 2014; Cui et al., 2015). Notably, PRG4 expression is affected by varying magnitudes of mechanical loading (Neu et al., 2007; Ni et al., 2012). An appropriate intermittent hydrostatic pressure (4 h per day for 2 days at 100 kPa) was able to upregulate PRG4 and prevent tumor necrosis factor α (TNFα)-mediated PRG4 inhibition in rat synovial fibroblasts (Xu et al., 2012), and PRG4 may play a compensatory role under abnormal mechanical stimulation. In a rat model of mandibular lateral shift, PRG4 was upregulated, the thickness of the superficial layer was increased, and the matrix-degrading activity of condylar cartilage was not obvious (Yang W. et al., 2020).

Superficial zone protein (SZP) and lubricin are homologous to PRG4 (Flannery et al., 1999) and have similar proliferative properties to those of PRG4. One study showed that moderate mechanical stimulation (7% elongation cyclic tensile strain) enhanced expression of SZP by upregulating transforming growth factor-beta (TGF-β) in condylar chondrocytes, whereas excessive mechanical stimulation (21% elongation cyclic tensile strain) inhibited synthesis of SZP by upregulating interleukin-1 beta (IL-1β) (Kamiya et al., 2010). In another study, the authors found that functional mandibular forward repositioning elevated lubricin expression, which is responsible for an excellent mechanical environment, maintaining the function and remodeling of the rat condyle and mandible (Chen Z. et al., 2019).

### 4.2 Promotion of chondrocyte proliferation

The proliferative capacity of chondrocytes decreases with age, which is one of the major difficulties in repairing degenerative cartilage (Hou et al., 2018). Moderate mechanical loading can facilitate cellular proliferation. Liu et al. developed a bilateral anterior elevation (BAE) mouse model in which the intra-articular space of the TMJ was gradually increased, with distraction and elongation loading on condylar cartilage. Moreover, loading upregulated expression of Cyclin D1, increased cell number and rendered condylar cartilage thicker. *In vitro,* 6% CTS upregulated Cyclin D1 in chondrocytes obtained from superficial and deep zone, also suggesting that moderate mechanical loading promotes cell proliferation (Liu Q. et al., 2019).

High-mobility group box 2 (HMGB2) is involved in mechanical force-induced cell proliferation. In the BAE mouse model, HMGB2 upregulation was observed in thickened condylar cartilage. The authors confirmed *in vitro* that under negative pressure, HMGB2 was upregulated in superficial condylar chondrocytes and stimulated proliferation via activation of the AKT signaling pathway (Liu et al., 2021b).

Insulin-like growth factor I (IGF-I) has been confirmed to regulate the proliferation and differentiation of rat condylar cartilage progenitor cells (Fuentes et al., 2002). In rats treated with a mandibular propulsive appliance (an appliance that exerts mechanical loading on the condylar cartilage), upregulation of IGF-I and IGF-II as well as proliferating cell nuclear antigen (PCNA) in the condylar cartilage was observed (Hajjar et al., 2003). *In vitro*, 7% elongation stretching increased expression of IGF-I, IGF-II and PCNA, indicating that IGFs mediate the cell proliferation induced by mechanical stimulation (Marques et al., 2008).

### 4.3 Maintenance of cartilage integrity

Cartilage consists of chondrocytes as well as the abundant proteoglycans and collagen in the ECM. The collagen network confers tensile strength, and proteoglycans aggregate to resist compressive force (Haleem-Smith et al., 2012). A moderate degree of mechanical loading can enhance cartilage matrix synthesis and maintain normal structure and function. Aggrecan in condylar chondrocytes was upregulated under a low pressure of 90 kPa for 60 min, and expression of the proinflammatory prostaglandin F1α was inhibited (Chen et al., 2007). Expression of Collagen II in condylar cartilage was enhanced under functional loading (Rabie et al., 2003a). Sun et al. further explored the underlying mechanism involved and found that both Collagen II and Raf kinase inhibitor protein (RKIP) were upregulated in the condylar cartilage of rats in the mandibular advancement group. Then, they demonstrated *in vitro* that the expression levels of aggrecan and Collagen II increased gradually with the duration of CTS (16%, 1 Hz), accompanied by inhibition of ERK signaling. This phenomenon was reversed after RKIP knockdown, suggesting that moderate mechanical stimulation enhances matrix secretory activity by upregulating RKIP and inhibiting the ERK pathway (Sun et al., 2017).

Moderate mechanical loading can also exert anticatabolic and anti-inflammatory effects, ideally by impeding cartilage damage. CTS (6%) has been confirmed to reduce expression of MMP13 in condylar chondrocytes stimulated with IL-1β or TNFα (Tabeian et al., 2017; Tabeian et al., 2019). In addition, low magnitudes of CTS ranging from 3% to 9% inhibit rHuIL-1β-induced nitric oxide (NO) production, though higher magnitudes of CTS (12%) do not demonstrate anti-inflammatory effects, indicating that different magnitudes of mechanical loading may exert different effects on condylar chondrocytes (Agarwal et al., 2001). Furthermore, an *in vivo* study confirmed that replacement of abnormal UAC with moderate BAE in mice rescued condylar cartilage degeneration (Zhou P. et al., 2020).

### 4.4 Promotion of endochondral ossification

Adaptive remodeling of mandibular condylar cartilage is strongly affected by mechanical loading and constitutes the primary basis for orthodontic functional therapy (Basdra et al., 1994; Shen and Darendeliler, 2005). Endochondral ossification is the core process of adaptive remodeling. Specifically, mesenchymal stem cells (MSCs) in cartilage are induced to differentiate into chondrocytes under external stimuli, followed by an increase in the population of proliferating chondrocytes. Subsequently, these chondrocytes mature into a hypertrophic phenotype that undergoes terminal differentiation and also synthesize an ECM abundant in type X collagen; moreover, neovascularization is increased, which recruits osteoblasts and initiates osteogenesis in cartilage (Kronenberg, 2003; Shen and Darendeliler, 2005). To better understand the mechanism behind endochondral ossification upon moderate mechanical loading, several critical signaling molecules deserve attention. We discuss them according to different biological functions.

#### 4.4.1 Increasing new chondrocyte populations

Increased numbers of chondrocytes enhance synthesis of the cartilage matrix, providing a template for bone formation (Rabie and Hägg, 2002). Parathyroid hormone-related protein (PTHrP) is known to limit the speed of chondrocyte maturation and differentiation (Amling et al., 1997). One study demonstrated that mandibular advancement in rats induced MSC differentiation into chondrocytes and stimulated PTHrP expression, thereby delaying subsequent chondrocyte maturation and allowing additional chondrocyte generation (Rabie et al., 2003b). SOX9 is another factor capable of enhancing differentiation of MSCs into chondrocytes (Dy et al., 2012). Advancement of the mandible upregulates SOX9, which induces more MSCs to differentiate into chondrocytes, followed by increased cartilage matrix synthesis (Rabie et al., 2003a). In addition, Ng et al. reported that SOX9 and PTHrP exhibited similar expression patterns under repeated mechanical loading induced by a bite-jumping appliance (Ng et al., 2006).

#### 4.4.2 Promotion of chondrocyte maturation and differentiation

Chondrocyte maturation and differentiation are essential for subsequent endochondral ossification. Expression of RUNX2, which mediates chondrocyte terminal maturation and hypertrophic mineralization, has been detected in the mandibular condyle (Rabie et al., 2004; Ding et al., 2012). In a rat model of mandibular advancement, *Runx2* mRNA was highly expressed in the condylar cartilage and subchondral bone, and expression of Collagen X was elevated, indicating enhancement of terminal maturation, facilitating endochondral ossification (Tang and Rabie, 2005). Moreover, decreased mineralization correlates with reduced expression of RUNX2 in condylar cartilage on the low mechanical loading side (Dutra et al., 2018).

The activator protein-1 (AP-1) transcription factor also plays an important role in promoting chondrocyte maturation and differentiation (Thomas et al., 2000). In rats fed a hard diet, AP-1 proteins, including Fra-1, Fra-2, JunB and JunD, exhibited greater expression than in the soft diet group throughout all stages of condylar cartilage differentiation (Papachristou et al., 2006). In addition, AP-1 proteins have been reported to trigger subsequent biochemical responses by interacting with Runx2 and forming complexes (D’Alonzo et al., 2002). Dionysios et al. demonstrated that pc-Jun, c-Fos, JNK2, p-JNK, p-ERK and Runx2 were upregulated in the condylar cartilage of hard diet rats, suggesting that mechanical loading activated the AP-1 and Runx2 transcription factors via the c-Jun N-terminal kinase (JNK) and ERK MAPK signaling pathways (Papachristou et al., 2005).

#### 4.4.3 Regulation of angiogenesis

The mandibular condylar cartilage is a tissue without lymphatic or vascular networks. Angiogenesis facilitates influx of circulating factors that stimulate replacement of hypertrophic cartilage matrix with bone, indicating the onset of endochondral ossification (Harper and Klagsbrun, 1999). Vascular endothelial growth factor (VEGF) is the primary mediator governing vascular development and angiogenesis and is regarded as a promising candidate for promoting chondrocyte maturation and apoptosis, ECM remodeling, neovascularization and recruitment of osteoblast progenitors (Gerber et al., 1999; Jiang et al., 2017). VEGF has been found to be upregulated in the condylar cartilage of rats fed a hard diet along with activation of the p44/42 MAPK and p38 MAPK signaling pathways (Jiang et al., 2017). In addition, increased production of VEGF and condylar bone was observed at the later stages of stepwise advancement (Leung et al., 2004). The above studies indicate that moderate mechanical stimulation promotes the formation of condylar bone by upregulating VEGF and activating the MAPK signaling pathway.

Angiopoietin (Ang) is also involved in angiogenesis. The autocrine Ang-1/Tie-2 signaling pathway regulates the plasticity of blood vessels and plays a role in the maintenance of vascular integrity (Cascone et al., 2003). Ang-2 functions as an endogenous inhibitor of Ang-1, blocking activation of Tie2 induced by Ang-1. Upregulation of Ang-2 expression serves as an early indicator of angiogenesis, as it facilitates early vascular degeneration and promotes angiogenesis (Oike et al., 2004). In a rabbit model with forward mandibular positioning, Ang-1 and Ang-2 were shown to be upregulated with chondrocyte maturation, especially in the hypertrophic layer, suggesting that Ang-1 and Ang-2 may play a role in stimulating angiogenesis within the hypertrophic layer of the condylar cartilage (Jing et al., 2013).

Notably, even if moderate levels of VEGF are essential for the coupling of angiogenesis and osteogenesis, abnormal mechanical stress can lead to VEGF overexpression, accompanied by subchondral bone loss and enhanced catabolism of chondrocytes, which may contribute to the initiation and progression of TMJOA (Jiao et al., 2011; Farias-Neto et al., 2012; Grosso et al., 2017).

## 5 Abnormal mechanical stimulation

**FIGURE 3:**
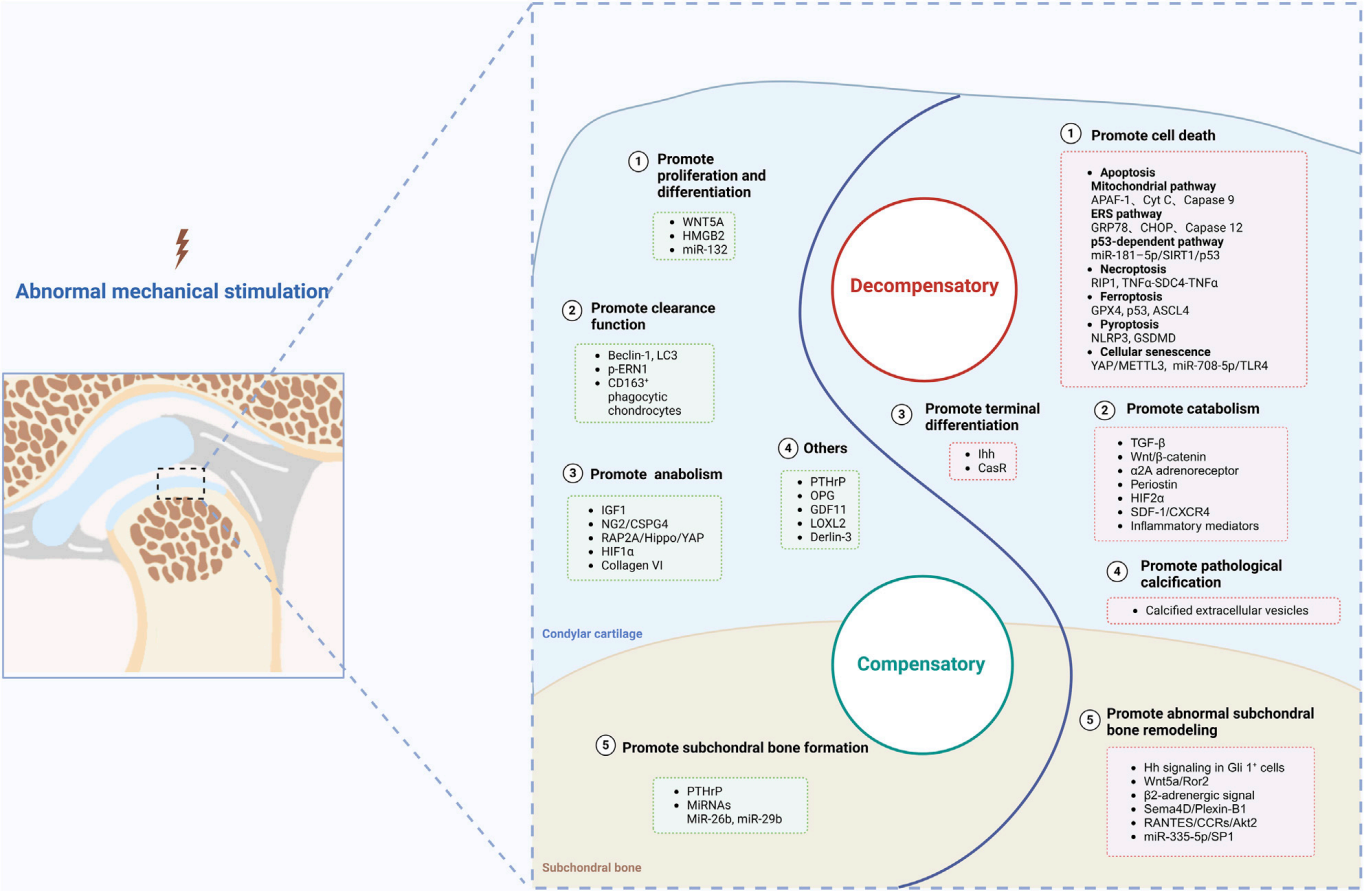
Molecules with compensatory and decompensatory effects under abnormal mechanical stimulation. Created with BioRender.com.

Under abnormal mechanical stimulation, the repair capacity of TMJ condylar cartilage is impaired, which means that the balance of cartilage homeostasis is disrupted, accompanied by enhanced catabolic activities. Eventually, this imbalance results in cartilage degeneration and promotes the progression of TMJOA. During the occurrence and development of TMJOA, critical signaling molecules may play compensatory or decompensatory roles; thus, we classify these molecules into two main categories: molecules with compensatory or decompensatory effects (Figure 3).

### 5.1 Molecules with compensatory effects

Notably, at the early stage of abnormal stimulation, several molecules are upregulated to maintain cartilage homeostasis. In the decompensation stage, there are still several upregulated molecules that are involved in repair of cartilage tissue, even though they eventually fail. Moreover, although expression of several molecules is downregulated upon abnormal mechanical stress, if certain interventions are applied, their expression will increase to impede OA progression. We call these molecules compensatory molecules with compensatory effect and discuss them in five main directions.

#### 5.1.1 Molecules that promote condylar chondrocyte proliferation and differentiation

To ameliorate cartilage degeneration, chondrocytes attempt to increase proliferation and remodeling at the beginning of osteoarthritis (Goldring, 2012). In this section, we discuss the molecules capable of promoting proliferation and differentiation in chondrocytes upon treatment with excessive mechanical stimulation.

WNT5A belongs to the noncanonical class of Wnt family proteins and activates independent signaling pathways instead of a β-catenin-dependent pathway. An earlier study reported that WNT-5A can modulate chondrocyte proliferation and differentiation while inhibiting maturation (Yang et al., 2003). Similarly, Ge et al. demonstrated that expression of WNT5A was strongly increased in rat condylar cartilage at the early stage of mouth-opening-induced TMJOA, which facilitated proliferation, hypertrophy and migration via upregulation of c-MYC and Cyclin D1 via the JNK signaling pathway, suggesting the role of WNT5A in repairing condylar cartilage (Ge et al., 2017).

HMGB2 and β-catenin are important transcriptional regulators that regulate chondrocyte proliferation and differentiation. HMGB2, a DNA-binding protein widely distributed in chromatin and dominantly expressed in the superficial zone of articular cartilage, is reported to regulate chondrocyte survival through the Wnt/β-catenin pathway (Taniguchi et al., 2009; Starkova et al., 2023). Under hydrostatic pressure, the Wnt/β-catenin pathway in chondrocytes is activated. Then, the stabilized β-catenin protein is shuttled to the nucleus and regulates downstream gene transcription, eventually promoting cell proliferation (Cheleschi et al., 2017). Zhou et al. discovered that silencing HMGB2 expression renders condylar chondrocytes insensitive to hydrostatic pressure loading, resulting in a significant decrease in β-catenin. They further found in a rabbit model of surgical anterior disc displacement that expression levels of HMGB2 and β-catenin were upregulated during the first week, which promoted the differentiation and maturation of chondrocytes in the fibrous and proliferative layers. These findings indicate that crosstalk between HMGB2 and β-catenin exists (Zhou Y. et al., 2020).

The level of miR-132 in plasma from OA patients has been found to be significantly lower than that in healthy controls (Murata et al., 2010). Zhou et al. discovered that expression of miR-132-3p in condylar cartilage was aberrantly downregulated in a UAC rat model. IL-1β-induced condylar chondrocytes also exhibited low miR-132-3p expression. Following miR-132-3p overexpression, cellular proliferation activity and matrix synthesis improved, and apoptosis and inflammatory responses were inhibited (Zhou et al., 2022).

#### 5.1.2 Molecules that promote clearance function

Autophagy and phagocytosis are two conserved endogenous lysosomal dependent clearance processes that degrade harmful intracellular and extracellular material and are necessary for maintaining cellular and tissue homeostasis (Bonilla et al., 2013).

Autophagy, which is responsible for the degradation of impaired membranes, organelles and macromolecules, has been reported to be an intracellular protective mechanism for maintaining cartilage homeostasis (Martínez-Borra and López-Larrea, 2012). Beclin-1 and LC3 are two critical factors for the formation and expansion of autophagosomes (Shpilka et al., 2011; Li et al., 2012). In an experimentally induced disordered occlusion model in rats, upregulation of Beclin-1 and LC3 and decreased expression of mTOR (an inhibitor of autophagy) and mitogen-activated protein kinase kinase 3 (MAP4K3) (a regulator of mTOR) were observed in both the hypertrophic and proliferative layers of condylar cartilage, suggesting enhanced autophagy under abnormal mechanical stimulation (Zhang et al., 2013).

Notably, autophagy can be mediated by endoplasmic reticulum stress (ERS). Endoplasmic reticulum to nucleus signaling 1 (ERN1), activating transcription factor 6 (ATF6) and eukaryotic translation initiation factor 2 alpha kinase 3 (EIF2AK3) are the three main ER transmembrane proteins associated with ERS. In addition, the mechanistic target of rapamycin complex 1 (MTORC1) signaling pathway has been reported to be a key regulator of autophagy (Han et al., 2022). Yang et al. demonstrated in flow fluid shear stress (FFSS)-treated chondrocytes that MTORC1 promotes p-EIF2AK3-mediated ERS-related apoptosis while inhibiting autophagosome formation. In addition, p-ERN1 was confirmed to be the upstream molecule of MTORC1 that exerted inhibitory effects on MTORC1 to suppress ERS-related apoptosis and promote autophagy. The authors further observed in a UAC rat model that autophagy mediated by p-ERN1 and ERS-related apoptosis mediated by p-EIF2AK3 were activated simultaneously at the early stage meanwhile MTORC1 was suppressed. However, at the late stage, expression of p-ERN1 decreased gradually, followed by release of MTORC1, which resulted in a transition from protective autophagy to prodeath ERS-related apoptosis, accelerating the progression of TMJOA (Yang H. et al., 2020).

The expression level of cluster of differentiation 163 (CD163) is regarded as a functional indicator of nonprofessional phagocytes (Castillo and Kourí, 2004). Jiao et al. identified CD163^+^ phagocytic chondrocytes in the cartilage of healthy knees and TMJs from SD rats for the first time. In their study, TMJOA-like lesions were induced by experimentally induced disordered occlusion, and many CD163^+^ chondrocytes exhibited active phagocytic and migratory capabilities to facilitate elimination of degraded cartilage tissue and impede the TMJOA progression. Interestingly, it is known that the proper degree of ECM degradation can promote the mobilization of CD163^+^ chondrocytes, but this degradation leads to decreased nutrient availability; thus, phagocyte viability cannot be maintained (Jiao et al., 2013). Hence, the scavenger function of CD163^+^ chondrocytes is very limited.

#### 5.1.3 Molecules that promote anabolism of condylar cartilage

At present, more studies have focused on catabolism caused by aberrant mechanical stimulation, whereas anabolism has been ignored. In fact, both anabolism and catabolism are activated throughout the progression of TMJOA. Whether progression is aggravated depends on the balance between anabolism and catabolism. Even in degenerating cartilage, anabolism can be activated, attempting to protect against catabolic actions for the maintenance of cartilage integrity. Therefore, anabolism and associated signaling molecules promoted by abnormal mechanical loading deserve further attention (Sandell, 2007).

IGF1 is considered a crucial anabolism factor in cartilage and has been confirmed to promote biosynthesis of type II collagen and proteoglycans (Schneiderman et al., 1995; Mullen et al., 2015). Moreover, IGF1 can protect chondrocytes from apoptosis and stimulate proliferation (Higgins and Johnson, 2010; Wei et al., 2017). These physiological effects of IGF1 are initiated through its specific binding to transmembrane receptors, known as insulin-like growth factor 1 receptor (IGFR1) (Schmal et al., 2014). In a rat model of molar malocclusions, expression levels of IGF1, IGFR1 and Collagen II were elevated to promote repair of OA-like degenerative lesions (Yu et al., 2012). Notably, binding of IGF1 to IGFR1 is tightly controlled by extracellular IGF-binding proteins (IGFBPs) (Jones and Clemmons, 1995). IGF1 has a greater affinity for IGFBP than IGFR1. In a UAC rat model, Wang et al. reported that highly expressed IGFBP-3 and -5 competitively bind to IGF1, therefore attenuating IGF1 biological activity. Thus, injecting IGF1 might enhance IGF1R-mediated signaling, promote anabolism and impede mechanically stimulated progressive degeneration of condylar cartilage (Wang et al., 2019).

Neuron-glial antigen 2 (NG2)/chondroitin sulfate proteoglycan 4 (CSPG4) reportedly increases anabolism via the ERK 1/2 signaling pathway. NG2/CSPG4 is a transmembrane glycoprotein of the N-linked type I that binds to pericellular collagen VI and is characterized by the presence of chondroitin sulfate proteoglycans on its ectodomain (Burg et al., 1996). Reed et al. induced TMJOA by unilateral partial discectomy in both control and NG2/CSPG4 knockout mice. Expression of NG2/CSPG4 in control mice was significantly reduced at the early stage. In addition, upregulation of matrix degradation-related genes such as ADAMTS5 and MMP13 as well as of proinflammatory C-C motif chemokine ligand 2 (CCL2)/monocyte chemoattractant protein 1 (MCP1) and downregulation of matrix synthesis-related genes such as Col6a1, platelet-derived growth factor receptor beta (PDGFrβ) and TGF-β were detected in NG2/CSPG4 knockout mice. The above results prove the ability of NG2/CSPG4 to enhance anabolic metabolism (Reed et al., 2022). The ERK 1/2 signaling pathway has been extensively characterized as a mediator of mechanical loading across diverse cell types, controlling essential cellular processes, including proliferation, differentiation and cell survival (Ghilardi et al., 2020). The authors further demonstrated that NG2/CSPG4 knockout condylar chondrocytes exhibited a significant reduction in total and phosphorylated ERK 1/2 under static compression *in vitro* and in a TMJOA model *in vivo* (Reed et al., 2022). Hence, NG2/CSPG4 is an essential regulator of cartilage homeostasis associated with the ERK 1/2 signaling pathway. In summary, enhancing expression levels of NG2/CSPG4 might improve anabolic metabolism and potentially impede cartilage degeneration.

The Hippo/YAP pathway transduces mechanical stress stimulation to regulate chondrocyte anabolic capacity and phenotype (Maurer and Lammerding, 2019). In general, a rigid ECM or excessive mechanical stress induces inactivation of the Hippo pathway, resulting in dephosphorylation of YAP (activation of YAP) and enabling its translocation to the nucleus. Then, YAP forms complexes with transcription factors to control expression of downstream genes, which is strongly associated with the pathological processes of OA (Deng et al., 2018; Totaro et al., 2018; Ma et al., 2019). Ras-related protein Rap-2a (RAP2A) is a kind of small GTPase (Meng et al., 2018). Recently, RAP2A was verified to be a mechanotransduction molecule involved in TMJOA progression. The study showed that expression of RAP2A decreased with UAC-induced cartilage degeneration in TMJOA mice. A typical TMJOA phenotype (thinner cartilage layer, fewer chondrocytes and decreased expression of COL2A1 and proteoglycans) was observed in RAP2A knockout mice. Moreover, inactivation of the Hippo pathway and activation of YAP were observed, indicating that the RAP2A/Hippo/YAP pathway may play a critical role in regulating condylar cartilage homeostasis. Overexpression of RAP2A by Ad-Rap2a-GFP and inhibition of active YAP by verteporfin reinstated the normal phenotype and anabolic function of chondrocytes. Therefore, the RAP2A/Hippo/YAP pathway may play a critical role in regulating condylar cartilage anabolism, and targeting RAP2A or YAP might be a treatment option for TMJOA (Qi et al., 2022a).

Hypoxia-inducible factor 1α (HIF1α) is considered an anabolic factor that mediates upregulation of SOX9, COL2A1 and aggrecan under hypoxic conditions (Sanz-Ramos et al., 2013). Due to a lack of blood vessels and nerves, condylar cartilage is maintained in a hypoxic environment. Under abnormal dental occlusion force, the hypoxic condition in condylar cartilage is aggravated, and HIF1α is upregulated at an early stage to prevent articular cartilage degeneration. However, with continuous abnormal occlusion stress stimulation, expression of HIF2α, a catabolic factor, increases gradually, which acts as a negative feedback loop on HIF1α, accelerating condylar cartilage degeneration (Zhang et al., 2022b).

Collagen VI is one of the major components of the pericellular matrix (PCM), which is a thin layer of ECM that surrounds chondrocytes tightly and can not only transduce biochemical and biomechanical signals but also maintain the chondrocyte phenotype and structural integrity (Hing et al., 2002; Guilak et al., 2006). Chu et al. confirmed that collagen VI attenuated catabolism under IL-1β stimulation (Chu et al., 2017). A recent study showed that expression of collagen VI was increased in the condylar cartilage of rats under overloading conditions, indicating that chondrocytes attempt to enhance synthesis of collagen VI to maintain PCM integrity (Franklin et al., 2022).

#### 5.1.4 Other molecules with compensatory effects on condylar cartilage

In addition to the abovementioned molecules, compensatory molecules can play a role in inhibiting terminal differentiation, apoptosis and aberrant lipid metabolism as well as promoting matrix crosslinking.

PTHrP has been reported to prevent chondrocyte terminal differentiation directly (Zerega et al., 1999). A recent study showed that PTHrP expression tended to increase at the early stage of occlusal elevation-induced TMJOA but that expression of Collagen X, a marker of chondrocyte hypertrophy, was downregulated. However, expression of parathyroid hormone receptor 1 (PTH1R) (the sole receptor for PTHrP) decreased gradually. Hence, during the progression of TMJOA, PTHrP was unable to protect condylar cartilage effectively. The authors noted that the mismatch in expression of PTHrP and PTH1R may be one of the factors that initiates TMJOA (Zhuang et al., 2023).

Osteoprotegerin (OPG) has been reported to be a decoy receptor for receptor activator of nuclear factor kappa-B ligand (RANKL) that effectively competes with RANK, thereby attenuating the signaling cascade responsible for osteoclast and chondroclast activation (Edwards and Mundy, 2011; Knowles et al., 2012). OPG also exerts a protective effect on condylar cartilage by inhibiting chondrocyte apoptosis. In a rat model of hyperocclusion, expression of OPG was found to increase after 4 weeks. The authors further demonstrated that in OPG-knockout mice, more apoptotic condylar chondrocytes were detected, and TMJOA progression was accelerated (Chen D. et al., 2019). Moreover, Derlin-3 has been reported to suppress ERS-mediated apoptosis. Liu et al. confirmed the protective role of Derlin-3 in a UAC mouse model. Their data indicated that expression of Derlin-3 was upregulated at the early stage but decreased at the late stage. The decrease in Derlin-3 expression induced by UAC was associated with enhanced ERS-mediated apoptosis in degenerative condylar cartilage, which was reversed by removal of UAC (Liu et al., 2020).

Abnormal lipid metabolism has been proven to be involved in the incidence and progression of OA (Aspden et al., 2001). Growth differentiation factor 11 (GDF11) has been confirmed to inhibit the adipogenic differentiation of bone marrow mesenchymal stem cells and to decrease lipid accumulation in monocytes and hepatocytes (Luo et al., 2019; Hernandez et al., 2021). In the skeletal muscle tissue of obese mice, the expression level of GDF11 was downregulated (Egerman et al., 2015). After FFSS stimulation of primary condylar chondrocytes or UAC stimulation in mice, expression of GDF11 was inhibited, resulting in aberrant adipogenesis. In addition, supplementation with exogenous GDF11 alleviated degenerative changes (Wang H. et al., 2022).

LOXL2, a member of the lysyl oxidase (LOX) protein family, serves as an extracellular enzyme that promotes crosslinking of collagen and elastin within the ECM. Consequently, LOXL2 plays a pivotal role in enhancing tensile strength and maintaining the structural integrity of condylar cartilage (Csiszar, 2001). Zhang et al. discovered that expression of LOXL2 was decreased in a rat model of TMJOA induced by compressive mechanical force. After injection of recombinant LOXL2 (rhLOXL2), degenerative condylar cartilage was rescued with restoration of proteoglycans and collagen II, highlighting the role of LOXL2 in facilitating matrix crosslinking (Zhang et al., 2021).

#### 5.1.5 Promotion of subchondral bone formation

Due to the unique mechanical properties of condylar cartilage, the alterations induced by continued abnormal loading affect the distribution of stresses and strains in the subchondral layers, which mediates short-term damage and long-term subchondral bone remodeling processes (Kuroda et al., 2009). Moreover, generation of microfractures and microcracks at the osteochondral interface leads to enhanced crosstalk between cartilage and subchondral bone, which further exacerbates cartilage degeneration and subchondral bone loss (Liu et al., 2021a). In this section, we focus on molecules that promote subchondral bone formation and inhibit bone loss.

PTHrP, as a protective factor, is capable of binding to PTH1R located on the cell membrane of osteoblasts, subsequently triggering cascade signaling pathways to effectively modulate bone metabolism (Jolette et al., 2017). Zhang et al. reported that after subcutaneous injections of intermittent PTHrP (iPTH) in rats with occlusal disorders, condylar cartilage degeneration was alleviated and that this change was accompanied by an improvement in subchondral bone formation. These authors confirmed that iPTH increased the osteoblastic differentiation potential of condylar subchondral bone marrow-derived mesenchymal stem cells (SMSCs) and inhibits phosphorylation of Smad2/3, which indicates inhibition of TGF-β signaling (Zhang et al., 2022c). Increased expression of TGF-β has been found in the condylar cartilage and subchondral bone of both aging mice and rats with disordered occlusion, suggesting that dysregulated activation of the TGF-β signaling pathway may serve as a critical factor in the pathogenesis of TMJOA (Zheng et al., 2018). Taken together, these findings suggest that PTHrP alleviates cartilage deterioration and improves subchondral bone remodeling by enhancing SMSC osteoblastic differentiation and suppressing activation of TGF-β signaling (Zhang et al., 2022c).

MiR-26b and miR-29b are reported to promote osteogenic differentiation (Trompeter et al., 2013). Yang et al. discovered that expression of miR-26b was significantly downregulated in subchondral BMSCs of UAC rats and upregulated during the process of osteogenesis. Overexpression of miR-26b in condylar subchondral bone promoted osteogenesis and rescued bone loss through activation of β-catenin. Notably, the increase in miR-26b in BMSCs markedly alleviated cartilage degeneration (Yang et al., 2022). Similarly, decreased miR-29b expression was observed in a UAC mouse model. In addition, intra-articular treatment with aptamer-agomiR-29b rescued the deterioration of condylar cartilage and subchondral bone as well as the hyperfunction of osteoclasts (Sun et al., 2020a).

### 5.2 Molecules with decompensatory effects

It is difficult for molecules with compensatory effects to maintain homeostasis with extended duration of abnormal mechanical stimulation, and molecules with decompensatory effects begin to occupy a dominant position during TMJOA progression, resulting in gradual disruption of the balance. Molecules with decompensatory effects play a promotive role mainly in cell death, catabolism, terminal differentiation, pathological calcification and abnormal subchondral bone remodeling. We discuss them in five directions subsequently.

#### 5.2.1 Promotion of cell death

Due to the avascular nature of the cartilage matrix and limited proliferative capacity of chondrocytes, massive numbers of cells die, contributing to cartilage degeneration (Charlier et al., 2016). Several cell death processes such as apoptosis, necroptosis, ferroptosis, pyroptosis and cell senescence have been found to participate in TMJOA progression. Apoptosis is a type of programmed cell death and has been widely reported in studies of condylar cartilage stimulated by abnormal mechanical loading. In general, apoptosis pathways can be divided into two categories: the extrinsic pathway, also known as the death receptor pathway, and the intrinsic pathway, also known as the mitochondrial pathway (Elmore, 2007). Both exogenous and endogenous NO can activate mitochondria-dependent apoptosis (Maneiro et al., 2005). The level of NO is elevated in condylar chondrocytes after FFSS stimulation, which promotes permeability of the outer mitochondrial membrane, facilitating release of apoptotic factors such as apoptotic protease activating factor-1 (APAF-1), cytochrome C (Cyt C) and caspase-9 into the cytoplasm and then induces the chondrocyte apoptosis through the mitochondrial pathway (Ren et al., 2019). In addition, mechanical forces promote intrinsic mitochondria-dependent apoptosis mediated by ERS. Excessive ERS results in apoptotic events, which are mediated by the caspase-12-dependent pathway, the JNK pathway and C/EBP homologous protein (CHOP) (Ron and Walter, 2007; Tabas and Ron, 2011). One study showed that expression of glucose regulated protein 78 (GRP78) (a kind of ERS marker), CHOP and caspase-12 was upregulated in condylar chondrocytes under excessive hydrostatic pressure (HP) of 0.3 MPa, resulting in activation of ERS-mediated apoptosis (Xu et al., 2017). Another study confirmed that 20% of mechanical force promoted the apoptosis of condylar chondrocytes via upregulation of GRP78, GRP94 and caspase-12. Treatment with salubrinal (an ERS inhibitor) can impede apoptosis (Huang et al., 2017). Notably, a significant increase in cytoplasmic Ca^2+^ levels in condylar chondrocytes was observed under mechanical stress loading (Li et al., 2013). Zhu et al. confirmed that [Ca^2+^]i plays a critical role in mediating mechanical stress-induced ERS and subsequent apoptosis. However, they discovered that complete prevention of condylar chondrocyte apoptosis could not be achieved only through inhibition of Ca^2+^ signaling, indicating involvement of other apoptotic pathways in this process (Zhu et al., 2016). Several signaling molecules are involved in regulating apoptosis. Activation of silent information regulator 1 (SIRT1) induces p53 deacetylation, thereby inhibiting p53-dependent apoptosis (Xu et al., 2020). A recent study revealed that the expression level of miR-181a-5p was elevated in the condylar cartilage of UAC-induced TMJOA mice. The authors also demonstrated that miR-181a-5p directly targeted the 3’ untranslated region (UTR) of *Sirt1* and subsequently inhibited expression of SIRT1, promoting p53-dependent apoptosis. Therefore, the miR-181–5p/SIRT1/p53 axis facilitates chondrocyte apoptosis (Qi et al., 2022b).

Necroptosis, another form of programmed cell death, exhibits morphological features similar to those of necrosis. Unlike apoptosis, which induces cell death without disrupting the cell membrane, in necroptosis, the cell membrane is ruptured followed by release of intracellular contents (Linkermann and Green, 2014). Receptor interacting protein kinase 1 (RIP1) serves as a critical upstream regulator that mediates necroptosis (Liu Y. et al., 2019). Once the extracellular or intracellular balance is disrupted, activated RIP1 results in sequential activation of RIP3 and mixed lineage kinase domain-like protein (MLKL) (Galluzzi et al., 2018). Phosphorylated MLKL induces plasma membrane permeabilization and initiates the inflammatory response (Cho et al., 2009). It was reported that levels of RIP1, RIP3, and caspase-8 in condylar chondrocytes increase under compressive mechanical force in the 4-day group, indicating activation of necroptosis. However, the abovementioned factors returned to baseline levels after 7 days, which suggested that there were some adaptive mechanisms in chondrocytes; thus, cell death is a tightly controlled process restricted to the early stage of mechanical stress stimulation. Interestingly, the authors found that in contrast to caspase-8, which was expressed at increased levels throughout the cartilage, RIP1 was expressed at greater levels in chondrocytes at sites where mechanical force was applied, indicating that chondrocytes under more severe mechanical stimulation exhibit a greater propensity for necroptosis instead of apoptosis (Zhang et al., 2017). Furthermore, mechanical stimulation might induce a vicious necroptotic cycle. Damage-associated molecular patterns (DAMPs) are released from ruptured cells during necroptosis, which leads to more severe disruption of tissue homeostasis. Syndecan 4 (SDC4) has been identified as a DAMP. He et al. demonstrated a vicious necroptotic cycle of TNFα-SDC4-TNFα in a UAC rat model. Specifically, TNFα activated RIP3 and pMLKL in sequence, subsequently triggering necroptosis. In turn, the SDC4 released served as the key DAMP to enhance expression of TNFα, suggesting that a feedback loop further exacerbated necroptosis in chondrocytes and synoviocytes (He F. et al., 2022).

Ferroptosis is a novel form of programmed cell death triggered by intracellular accumulation of iron-dependent lipid peroxidation, which can be suppressed by glutathione peroxidase 4 (GPX4) while promoted by p53 and acyl coenzyme A synthetase long chain family, member 4 (ACSL4) (Yang and Stockwell, 2016; Maiorino et al., 2018; Delin et al., 2021). Cheng et al. observed decreased levels of GPX4 as well as increased levels of p53 and ASCL4 in the condyles of both occlusion disorder and UAC rat models. After injection of liproxstatin-1, a ferroptosis inhibitor, the condylar cartilage degradation was greatly rescued accompanied by upregulation of GPX4 and downregulation of p53 and ASCL4 (Cheng et al., 2023).

Pyroptosis is a type of pro-inflammatory programmed cell death that mediated by NOD-like receptor protein 3 (NLRP3) pyroptosome and gasdermin D (GSDMD) before rupture of plasma membrane (Chen et al., 2016; Huang Y. et al., 2021). A recent study confirmed chondrocyte pyroptosis in miodoacetate (MIA)-induced TMJOA mice (Xin et al., 2023). In another study, it was demonstrated that mechanical compression on the human hip joint cartilage could initiate the pyroptosis process and contribute to cartilage degradation, which was more serious under the 25 MPa compression than 15 MPa (Chunye et al., 2023). Therefore, we believe that chondrocyte pyroptosis in condylar cartilage can also be activated under excessive mechanical loading and then participate in TMJOA progression.

Cellular senescence is an irreversible biological phenomenon that characterized by a permanent growth arrest with a senescent-associated secretory phenotype (SASP), which secrets a large amount of proinflammatory factors (Bo et al., 2023). Thus, condylar chondrocyte senescence indicates a pathological change in TMJOA. It has been reported that cellular senescence is associated with N^6^-methyladenosine (m^6^A) modification that catalyzed by methyltransferase-like 3 (METTL3) (Xiulin et al., 2021). Yang et al. demonstrated that abnormal mechanical stimulation could induced chondrocyte senescence both *in vivo* (UAC-treated rats) and *in vitro* (20% CTS), which was partially attributed to the deficiency of YAP (a mechanosensitive element). They further confirmed that YAP deficiency enhanced expression of METTL3, thereby mediating m^6^A-dependent chondrocyte senescence (Yang et al., 2023). In addition, the role of microRNA in cellular senescence has attracted attention. miR-708-5p exhibits a positive correlation with the longevity of mice (Benjamin et al., 2017). A recent study showed that the expression of miR-708-5p exhibited a more significant decrease in condylar cartilage of UAC-treated adult rats compared to younger ones. Furthermore, the authors verified that toll-like receptor 4 (TLR4), which senses OA-related DAMPs, was the direct target of miR-708-5p and exogenous miR-708–5p could rescue senescence-like cell degeneration though inhibiting TLR4 expression (Lingfeng et al., 2024).

#### 5.2.2 Promotion of catabolism

##### 5.2.2.1 TGF-β

Transforming growth factor-beta (TGF-β) is a cytokine involved in various biological processes and plays an essential role in regulating cartilage homeostasis (Blaney Davidson et al., 2007). TGF-β1, a member of the TGF-β superfamily, appears to play dual roles in TMJOA. As reported, TGF-β1 can stimulate synthesis of proteoglycans in chondrocytes (van Beuningen et al., 1994) and suppress terminal differentiation of chondrocytes (Serra et al., 1997). Intra-articular injection of TGF-β1 effectively alleviated cartilage degeneration and protected subchondral cancellous bone in a TMJOA rabbit model induced by partial disc perforation (Ying et al., 2013).

However, other studies seemingly provided conflicting results. Aberrant elevation of TGF-β1 signaling was observed in a disordered occlusion rat model (Zheng et al., 2018). Overexpressed TGF-β1 can induce high temperature requirement A1 serine protease (HtrA1) generation, which contributes to excessive production of MMP13 (a marker of catabolism) (Long et al., 2016). Treatment with TGF-β1 initially induces proteoglycan synthesis; however, prolonged TGF-β1 exposure accelerates OA progression (Bakker et al., 2001). In a partial discectomy-induced TMJOA mouse model, conditional removal of transforming growth factor receptor type II (*Tgfbr*2) effectively attenuated condylar cartilage deterioration (Fang et al., 2017). Moreover, Embree et al. discovered that hyperactivated TGF-β1 stimulated chondrogenic differentiation and ECM synthesis in younger mice but led to ECM degradation and TMJOA in aging mice. Even over a prolonged duration, mandibular explant cultures subjected to low doses of TGF-β1 (2 ng/mL) did not exhibit notable changes in the hypertrophic zone area (Embree et al., 2010). Therefore, the effects of TGF-β1 on condylar cartilage depend on the exposure time, age and dosage.

##### 5.2.2.2 Wnt/β-catenin

The Wnt signaling pathway is crucial for regulating the growth, development and homeostasis of articular cartilage and is classified into two main categories: the canonical Wnt signaling pathway, which is dependent on β-catenin; and the noncanonical Wnt signaling pathway, which is independent of β-catenin (Li et al., 2023). Wnt/β-catenin is involved in the canonical Wnt signaling pathway. After active Wnt ligands bind to receptors on the cell membrane, the destruction complex is destabilized, releasing β-catenin. Then, free β-catenin translocates into the nucleus and binds to T-cell factor/lymphoid enhancer factor (TCF/LEF) transcription factors, which regulate expression of Wnt target genes (Zhou et al., 2017; Li et al., 2023).

Wnt/β-catenin signaling regulates chondrocyte proliferation, differentiation, hypertrophy and ECM synthesis (Stampella et al., 2017; Cheng et al., 2022). Appropriate levels of Wnt/β-catenin are critical for maintaining cartilage homeostasis and long-term function. Abnormal upregulation or downregulation of β-catenin in articular cartilage exacerbates OA (Cheng et al., 2022; Li et al., 2023). Overexpression of β-catenin triggers metalloproteinase production and chondrocyte hypertrophy, whereas low expression of β-catenin leads to chondrocyte death (Lories et al., 2013).

Nevertheless, the role of the Wnt/β-catenin pathway in excessive mechanical loading-induced TMJOA has not been determined. After compressive mechanical stress loading, Wnt/β-catenin signaling was inhibited in the condylar cartilage of rats. Moreover, activation of Wnt/β-catenin signaling promoted the proliferative capacity of condylar chondrocytes and alleviated cartilage degeneration (Jiang et al., 2018). In contrast, another study demonstrated that overloaded functional orthopedic force activated the Wnt/β-catenin signaling pathway, which contributed to condylar cartilage degeneration in rats. Moreover, sclerostin, which inhibits the Wnt/β-catenin signaling pathway, was downregulated (He Z. et al., 2022). These differences in expression may be attributed to differences in the modeling methods and durations of mechanical stimulation, but the detailed mechanism needs to be further explored.

In addition, the impact of noncanonical Wnt signaling pathways deserves attention. For example, Wnt16 may impede TMJOA progression through activating the Wnt/β-catenin signaling pathway (Hua et al., 2022). In fact, complex interactions occur among canonical and noncanonical cascades, Wnt antagonists and other signaling pathways and contribute to the maintenance of cartilage homeostasis (Monteagudo and Lories, 2017).

##### 5.2.2.3 α2A-Adrenoreceptor

Abnormal mechanical stimulation can mediate inflammatory processes through the norepinephrine/α2A-adrenoreceptor complex. Norepinephrine has been detected in synovial fluid from OA patients (Lorenz et al., 2016). In addition, expression of α2A-adrenoreceptor was elevated in condylar cartilage of UAC rats. Norepinephrine induced degenerative changes in cartilage and subchondral bone through the α2A-adrenoreceptor complex, and the α2-adrenoreceptor antagonist yohimbine inhibited the norepinephrine-induced increase in chondrocyte catabolic activities. Moreover, the authors confirmed *in vitro* that norepinephrine-α2A signals acted primarily through the ERK1/2 and protein kinase A (PKA) pathways, which stimulate production of MMP3, MMP13 and RANKL while inhibiting aggrecans expression (Jiao et al., 2016).

##### 5.2.2.4 Periostin

Periostin, a member of the fasciclin family, is an ECM protein that does not directly participate in ECM formation but has a dynamic function in facilitating cellular communication with the surrounding microenvironment and inducing specific effects (Zhu et al., 2021). Moreover, periostin has been identified as a critical mediator of the response to mechanical loading (Gerbaix et al., 2015). Periostin is highly expressed in human OA cartilage (Han et al., 2020). Attur et al. demonstrated that periostin played a catabolic role in OA cartilage by increasing MMP13 expression via the canonical Wnt signaling pathway (Attur et al., 2015). Fan et al. first detected expression of periostin in human TMJOA condylar cartilage. They further confirmed that excessive pressure loading upregulated periostin, which inhibited expression of collagen and proteoglycans by activating the nuclear factor kappa B (NF-κB) pathway and upregulating ADAMTS5 (Fan et al., 2020).

##### 5.2.2.5 HIF2α

Hypoxia-inducible factor 2α (HIF2α) has been identified as a critical regulator involved in the progression of OA and can directly upregulate expression of genes encoding catabolic factors (Yang et al., 2010). Overloaded cyclic tensile strain upregulated catabolic factors such as MMP3, MMP13 and ADAMTS4 and enhanced expression of HIF2α (Li et al., 2017). Moreover, inhibition of HIF2α expression downregulated MMP13 and ADAMT4 in condylar chondrocytes under cyclic compressive force stimulation, and interfering with the NF-κB/HIF2α pathway alleviated condylar cartilage degeneration in an occlusal trauma-induced TMJOA model (Li et al., 2022). In summary, abnormal mechanical stress can induce catabolism through the NF-κB/HIF2α pathway.

##### 5.2.2.6 SDF-1/CXCR4

Stromal cell-derived factor (SDF)-1, a member of the CXC subfamily of chemokines, modulates immune cell activation, differentiation, and migration through its interaction with its sole receptor CXC receptor 4 (CXCR4) (Liu et al., 2017; Kawaguchi et al., 2019). Several studies have reported that activation of the SDF-1/CXCR4 axis can promote catabolism and lead to cartilage degeneration but that inhibition of this axis alleviates this damage (Wang et al., 2016; Wang et al., 2020; Chen et al., 2022). Kuang et al. discovered that SDF-1 mainly existed in the subchondral bone marrow adjacent to the osteochondral interface under normal conditions and that CXCR4 was present at high levels in the hypertrophic layer of condylar cartilage. They further showed that the SDF-1/CXCR4 axis was activated and accompanied by upregulation of IL6 and MMP9 in a rat model induced by long-term experimentally induced malocclusion, which resulted in condylar cartilage destruction (Kuang et al., 2013). Recently, in a rat model of TMJOA induced by a mandibular advancement appliance, it was shown that subchondral bone destruction occurred earlier than cartilage degeneration. The increase in SDF-1 expression in osteoblasts promoted interaction of SDF-1 with CXCR4, followed by upregulation of MMP13, leading to the breakdown of cartilage. This process was attenuated after administration of the SDF-1 inhibitor ADM3100 (Yang J. et al., 2020).

##### 5.2.2.7 Inflammatory mediators

Cartilage matrix degradation caused by excessive mechanical stress can disturb the balance of proinflammatory and anti-inflammatory mediators, resulting in a state of low-grade inflammation, which plays a critical role in OA progression (Houard et al., 2013; Robinson et al., 2016; Molnar et al., 2021). Inflammatory mediators in the process of OA can reprogram chondrocytes into an ECM-catabolic state, promoting production of MMPs and ADAMTS and accelerating cartilage degeneration (Liu-Bryan and Terkeltaub, 2014; Robinson et al., 2016; Arra and Abu-Amer, 2023). Therefore, it is necessary to focus on changes in inflammatory mediators under abnormal mechanical loading.

Bromodomain containing 4 (BRD4) is regarded as a promising therapeutic target for numerous inflammatory disorders (Shi and Vakoc, 2014). Under compressive mechanical force in rats, BRD4 inhibition downregulated the expression of inflammatory mediators such as *Tnfα*, *Il-*1*β*, and *Il-*6 and alleviated condylar cartilage degeneration (Huang Z. et al., 2021). In addition, the author demonstrated that BRD4 functioned by promoting translation of triggering receptor expressed on myeloid cells 1 (TREM1).

The serum level of PGE2 is regarded as a marker of the inflammatory response in OA patients and plays a catabolic role in cartilage mainly through binding to prostaglandin E receptor 4 (EP4). Inhibition of EP4 can promote anabolism and inhibit catabolism (Vos et al., 2014; Jin et al., 2022). In a UAC-induced TMJOA rat model, the level of PGE2 in condylar cartilage was elevated, and this change was accompanied by catabolism (Zhang et al., 2014).

The receptor for advanced glycation end products (RAGE), which belongs to the immunoglobulin superfamily, is a cell-surface receptor expressed on a wide range of cell types. As an inflammatory mediator, RAGE interacts with ligands and induces a series of pro-inflammatory responses (Dong et al., 2022). The absence of RAGE inhibits MMP13 expression and attenuates TMJOA development, indicating the key role of RAGE in TMJOA progression (Matias et al., 2016).

Adipokines in adipose tissue have a proinflammatory effect, and leptin is the typical adipokine. Leptin can synergize with other cytokines to facilitate inflammatory reactions and accelerate the catabolic process in cartilage (Simopoulou et al., 2007; Issa and Griffin, 2012). In the condylar cartilage of mice subjected to excessive compressive mechanical force and a high-fat diet, leptin exhibited excessive expression, resulting in more severe TMJOA-like changes (Du J. et al., 2020).

#### 5.2.3 Promotion of terminal differentiation

Under physiological conditions, chondrocytes usually exhibit reduced proliferative potential and resist terminal differentiation. However, in the pathological state, chondrocytes proliferate progressively and initiate terminal differentiation, leading to a hypertrophic phenotype (increased expression of hypertrophic markers such as MMP13, collagen X and alkaline phosphatase (ALP), followed by initiation of apoptosis, focal calcification and vascularization (Dreier, 2010; Chawla et al., 2022). Eventually, cartilage homeostasis is disrupted, resulting in a series of degenerative changes. To better understand the mechanisms involved in TMJOA initiation and progression, in this section, we focus on the key signaling molecules that induce terminal differentiation.

Indian hedgehog (Ihh) proteins are members of the hedgehog family of proteins that play a critical role in TMJ development and are mainly affected by Patched1 (Ptch1) and Smoothened (Smo) (Mackie et al., 2011; Bechtold et al., 2019). Ihh binds to Ptch1 to inhibit Smo, which subsequently activates Gli zinc finger transcription factors (Gli). Then, Gli translocates to the nucleus, where it induces expression of the transcription factor *Runx2* and initiates chondrocyte hypertrophy (Lum and Beachy, 2004; Taschner et al., 2008; Amano et al., 2014; Sabol et al., 2018). Ihh, Smo, Ptch1 and Gli-1 can be effectively activated in condylar cartilage in response to bite-raising stimuli (Long et al., 2019).

Notably, there is a negative feedback loop between Ihh and PTHrP. Specifically, increased Ihh signaling facilitates chondrocyte terminal differentiation while promoting PTHrP secretion; conversely, persistent PTHrP signaling delays maturation of hypertrophic chondrocytes, thus resulting in a decrease in Ihh-secreting cells (Karp et al., 2000). However, under abnormal mechanical stimulation, more chondrocytes become hypertrophic, but inadequate target cells express PTHrP, suggesting disruption of the feedback loop. Thus, a decrease in the PTHrP concentration in cartilage may serve as an indicator of degenerative changes (Liu Q. et al., 2018). Aberrant activation of calcium-/CaMKII in proliferative chondrocytes enhances Ihh expression (Taschner et al., 2008). Liu et al. demonstrated that upregulation of CaMKII-Ihh signaling, along with subsequent disruption of the Ihh-PTHrP feedback loop, serves as the trigger for UAC-induced TMJOA lesions. Inhibition of initial CaMKII activation reversed impairment of the Ihh-PTHrP feedback loop and ameliorated biomechanically induced cartilage degeneration (Liu Q. et al., 2018).

In addition, the calcium-sensing receptor (CaSR) plays a critical role in promoting the terminal differentiation of chondrocytes. A study showed that FFSS stimulation enhanced ER Ca^2+^ loading and upregulated CaSR in condylar chondrocytes, which accelerated terminal differentiation without altering the extracellular Ca^2+^ concentration. The author further demonstrated that activation of CaSR reduced expression of PTH1R (the sole receptor for PTHrP) through a counteracting relationship, thereby promoting terminal differentiation (Zhang M. et al., 2019). Interestingly, in addition to inducing terminal differentiation in chondrocytes, a recent study revealed that CaSR plays a proliferative role in Prg4-expressing superficial zone cells under both BAE *in vivo* and FFSS treatment at 16 dyn/cm^2^ for 2 hours *in vitro* by regulating the PTHrP nuclear localization sequence instead of the PTH/PTHrP receptor signal, which indicates that CaSR may exert different effects on different states of chondrocytes (Zhou et al., 2023).

#### 5.2.4 Promotion of pathological calcification

Under abnormal stress, hypertrophic chondrocytes release matrix vesicles (MVs), which leads to pathological calcification of the ECM, a critical event in the early stage of cartilage degeneration. Mineral ions rapidly accumulate after calcification-competent MVs are released into the ECM, resulting in the formation of the initial crystalline phase within the luminal space of the vesicle. Upregulation of matrix-degrading enzymes can promote the process of mineral deposition by degrading the surrounding collagen fibers to enlarge the interfibrous space (Zhang et al., 2016; Yan et al., 2020). Calcified extracellular vesicles (EVs) are mainly derived from autophagosomes that express the microtubule-associated protein 1A/1B light chain 3B (LC3). Secretion of LC3-positive EVs can be attributed to disruption of autophagic flux, which is caused by histone deacetylase 6 (HDAC6)-mediated destabilization of microtubules. After intra-articular injection of tubacin (an HDAC6 inhibitor) in a UAC rat model, release of LC3-positive EVs was blocked, and pathological calcification of condylar cartilage and TMJOA progression were obviously alleviated (Yan et al., 2022).

Furthermore, a recent study detected numerous exosome-like structures in calcified cartilage under UAC stimulation. These chondrocyte-derived exosomes contained increased levels of calcification promoters, such as tissue-nonspecific alkaline phosphatase (TNAP), and decreased levels of inhibitors, such as the matrix Gla protein (MGP), thereby aggravating pathological calcification (Liu et al., 2022).

#### 5.2.5 Promotion of abnormal subchondral bone remodeling

In addition to cartilage degeneration, abnormal bone remodeling involving imbalanced bone formation and resorption is a hallmark of OA (Wang et al., 2015). Focusing on the critical signaling molecules that promote abnormal bone remodeling might help to elucidate the pathological molecular mechanism of TMJOA under excessive mechanical loading.

##### 5.2.5.1 Hh signaling in Gli 1^+^ cells

In a TMJOA mouse model induced by unilateral partial discectomy, Hh signaling was activated, resulting in excessive expansion of Gli 1^+^ cells accompanied by enhanced but irregular osteoblastic differentiation, which destroyed the microarchitecture in the subchondral bone and subsequently promoted TMJOA development. The authors confirmed that selective inhibition of Hh signaling could rescue destruction of subchondral bone and reduce inflammatory responses, indicating that Hh signaling in Gli 1^+^ cells is important for maintaining subchondral bone homeostasis (Lei et al., 2022).

##### 5.2.5.2 Wnt5a/Ror2

Wnt5a interacts with receptor tyrosine kinase-like orphan receptor 2 (Ror2) to activate the noncanonical Wnt signaling pathway, which negatively regulates skeletal homeostasis (Baron et al., 2012; Maeda et al., 2012). In a UAC rat model, Wnt5a/Ror2 signaling in BMSCs derived from TMJ subchondral bone was activated, which subsequently upregulated chemokine C-X-C motif ligand 12 (CXCL12) and RANKL. Ultimately, the migration and differentiation of osteoclast precursors were promoted along with enhanced osteoclast activity, leading to subchondral bone destruction. Moreover, the JNK and/or Ca^2+^/nuclear factor of activated T cells (NFAT) pathways was involved in this process (Yang et al., 2015).

##### 5.2.5.3 β2-Adrenergic signals

During the process of physiological bone remodeling, secretion of norepinephrine by sympathetic nerves inhibits bone formation while stimulating bone resorption, which is mediated mainly by the β2-adrenergic receptor (Adrb2) expressed by osteogenic cells (Elefteriou et al., 2014; Moriya et al., 2015). The β-adrenergic pathway serves as a major transmitter pathway in the bones of rats under mechanical loading (sseur et al., 2003). For the condylar subchondral bone, abnormal UAC stimulation triggered increased levels of sympathetic nerve fibers and norepinephrine, which activated the Adrb2-PKA pathway, prompting MSCs to secrete more RANKL, thereby exacerbating subchondral bone loss and enhancing osteoclastic activities (Jiao et al., 2015).

##### 5.2.5.4 Sema4D/Plexin-B1

The bone-forming ability of osteoblasts is dependent on their migration, differentiation and ability to express osteogenic-related factors. Elevated osteoblast motility has been demonstrated to inhibit the bone-forming activity of osteoblasts (Negishi-Koga et al., 2011). Semaphorin 4D (Sema4D) is a transmembrane protein expressed by osteoclasts, and Plexin-B1 is expressed by osteoblasts and is the specific receptor for Sema4D (Suzuki et al., 2008). Sema4D can bind to Plexin-B1 to inhibit bone formation by inducing osteoblast motility (Negishi-Koga et al., 2011). In a rat model of TMJOA induced by UAC, Sema4D and Plexin-B1 were upregulated in the subchondral bone at the early stage, and Sema4D promoted the migration of osteoblasts expressing Plexin-B1. Interestingly, increased mRNA expression levels of osteogenic-related factors, such as Runx2, alkaline phosphatase, osterix and osteocalcin, was also found. However, due to enhanced motility, the bone-forming ability of osteoblasts was impaired, eventually resulting in subchondral bone loss (Zhang et al., 2022d).

##### 5.2.5.5 RANTES/CCR/Akt2

Elevated levels of proinflammatory cytokines contribute to osteoclast differentiation and subsequent excessive bone resorption (Chow and Chin, 2020). RANTES, an inflammatory chemokine, can bind to chemokine receptors (CCRs) to recruit immune cells to sites of inflammation and promote osteoclast formation and bone destruction (Hampel et al., 2013; Feng et al., 2020). It was found that levels of RANTES were elevated in the synovial fluid of TMJOA patients (Feng et al., 2020). In addition, RANTES was upregulated significantly in condylar cartilage under excessive loading caused by disc displacement in rats, in which it further bound to CCRs, attracted macrophages to the osteochondral interface and activated the Akt2 pathway, resulting in aggressive subchondral bone loss (Feng et al., 2022).

##### 5.2.5.6 miR-335-5p/SP1

Endochondral ossification plays a crucial role in bone formation and development. Thus, impairment of this process may induce degenerative changes in subchondral bone (Long and Ornitz, 2013). Recently, Xia et al. reported increased levels of miR-335-5p and decreased levels of endochondral ossification-related genes in condylar cartilage samples from TMJOA patients, indicating a correlation between miR-335-5p and endochondral ossification. Then, in a UAC rat model, researchers demonstrated that activated miR-335-5p caused damage to endochondral ossification, leading to significant deterioration of trabecular bone, which was improved by treatment with antagomiR-335. Moreover, miR-335-5p inhibited endochondral ossification by directly targeting specific protein 1 (SP1) and activating the TGF-β pathway (Xia et al., 2023).

##### 5.2.5.7 Crosstalk between condylar cartilage and subchondral bone

In the physiological state, the osteochondral interface maintains a dynamic balance to adapt to the changing mechanical microenvironment (Liu et al., 2021a). However, under abnormal mechanical loading, the osteochondral interface thickens and stiffens along with impaired mechanical properties, which leads to the generation of microfractures and microcracks, thereby resulting in frequent crosstalk between cartilage and subchondral bone (Imhof et al., 2000; Zhang et al., 2018). Therefore, the signaling molecules expressed by chondrocytes may affect subchondral bone. For example, Kuang et al. demonstrated that disordered molar occlusion upregulated pro-osteoclastic factors such as SDF-1, RANKL, Wnt5a and TGF-β1 in rat condylar cartilage (Kuang et al., 2019). In addition, expression of the abovementioned molecules contributing to subchondral bone loss, such as RANTES, miR-335-5p and Erα, was upregulated in condylar cartilage, suggesting the occurrence of bone–cartilage interplay (Wu et al., 2019a; Feng et al., 2022; Xia et al., 2023). Similarly, subchondral bone cells can also affect chondrocytes. An *in vitro* study showed that under excessive cyclic tensile stress, osteoblasts derived from porcine mandibular condyles enhanced catabolism and inhibited anabolism in cocultured chondrocytes (Lin et al., 2010). Another study indicated that impact loading on the TMJ can directly enhance synthesis of IL-1β in subchondral bone, thereby promoting catabolism of the overlying cartilage (Lin et al., 2009). Adrb2 deletion in nestin^+^ MSCs in UAC mice not only attenuated subchondral bone loss but also impeded condylar cartilage degeneration and aberrant calcification (Sun et al., 2020b). Notably, abnormal remodeling compromises the capacity of subchondral bone to absorb and buffer stress and cannot provide adequate support to the overlying cartilage, thereby disrupting the biomechanical environment of chondrocytes, which also contributes to condylar cartilage degeneration (Mansell et al., 2007; Yuan et al., 2014; Lei et al., 2022).

## 6 Effects of mechanical loading on the synovial membrane and disc

In addition to chondrocytes, the synovial membrane and disc of the temporomandibular joint are regulated by mechanical loading. In this section, we discuss the influence of mechanical loading on the synovial membrane and disc under physiological or pathological conditions.

### 6.1 Synovial membrane

The synovial membrane is the major component of the temporomandibular joint and covers all intraarticular structures, except for the articular disc and articular cartilage of the eminence, fossa and mandibular condyle (Dijkgraaf et al., 1996). It is composed of two layers, including a synovial lining layer and a connective sublining layer. The major functions of synovial membrane are to produce synovial fluid components and provide nutrients for the condyle (Iwanaga et al., 2000; Nozawa-Inoue et al., 2003; Tanaka et al., 2008). There are two types of cells in the synovial membrane: macrophage-like type A cells and fibroblast-like type B cells (synovial fibroblasts, SFs) (Nagai et al., 2006). Previous research has largely focused on the effect of mechanical stresses on SFs. Therefore, in the following section, the influence of mechanical stresses on SFs in the temporomandibular joint is presented.

Under physiological conditions, the main function of SFs is to synthesize ECM and synovial fluid components (Wilkinson et al., 1992). Moderate stress is not harmful or even favorable. In a study by Nazet et al., short high-frequency tensile strain had no effect on matrix constituent production-related genes, such as *Col1a*1, *Col1a2*, hyaluronic acid synthase 1 (*Has*1), cell-migration-inducing hyaluronidase 2 (*Cemip*2), Fibronectin-1 (*Fn*1) and *Adamts*5 (Nazet et al., 2021; Nazet et al., 2022). Xu et al. reported that intermittent hydrostatic pressure loading increased accumulation of PRG4 (a secreted mucinous glycoprotein that is considered responsible for joint lubrication) and may be beneficial (Xu et al., 2012). However, the lubrication function is affected by excessive mechanical stress stimulation. The predominant protein family in the ECM is collagens, which mainly consist of alpha-1 and alpha-2 polypeptide chains. In SFs subjected to excessive tension, total collagen deposition was diminished. Hyaluronic acid (HA) is an important factor for joint lubrication. Excessive tensile strain treatment also decreased expression of glycosaminoglycan HA in SFs.

In addition to lubrication, SFs have been identified to be key players in inflammation-related processes. Moderate mechanical stress has only minor effects on inflammatory factor secretion or even decreases inflammatory mediator secretion in SFs. Gene expression of proinflammatory factors such as *Il-*6, intercellular adhesion molecule-1 (*Icam-*1)*, Cxcl-*1*, Cxcl-*2*, Il-*1*β*, and *Ptgs*2 is not affected by suitable tensile strain (Nazet et al., 2021). In another study, a moderate stretching strain reduced gene expression of *Il*6 and *Il*1*β* (Nazet et al., 2022). PGE2 is a principal mediator of inflammation in OA. In the study of Sambajon et al. (Sambajon et al., 2003), mechanical strain decreased PGE2 production in SFs. However, excessive mechanical stress might promote inflammation. Advanced strains led to increased expression of inflammatory factors, including *Cxcl-*1*, Cxcl-*2*, Ptgs*2, PGE2, IL-6 and IL-1b (Akamine et al., 2012; Nazet et al., 2021; Nazet et al., 2022). Harmful mechanical stimuli increased the level of elastin-derived peptides (EDPs), which further upregulated the expression of IL-6 and MMP12 (Kobayashi et al., 2017). HP increased expression of cadherin-11, which plays a critical role in evoking SF inflammatory factors that may contribute to synovial inflammation, cartilage degeneration and rheumatoid arthritis (Wu et al., 2013). Stretching stress increased the level of cyclooxygenase-2 (COX-2, involved in production of PGE2) and inducible nitric oxide synthase (iNOS, which synthesizes NO) via the NF-κB signaling pathway (Yamaza et al., 2003; Morisugi et al., 2010). MMPs can degrade the ECM and are expressed in the synovial fluid of osteoarthritic TMJs. Expression of MMP-1, -2, -3 and -9 is upregulated by excessive compression (Muroi et al., 2007; Akamine et al., 2012). IL-8 is an important cytokine for angiogenesis and a characteristic feature of the inflamed synovium. Excessive stress stimulates expression of IL-8 (Muroi et al., 2007; Akamine et al., 2012) and other angiogenic factors, including VEGF-D and FGF-2 (Wu et al., 2013).

In addition, SFs can mediate the bone remodeling process by secreting related proteins. Protein expression of RANKL in synovial cells treated with compressive force is increased, which further facilitates differentiation of osteoclasts (Ichimiya et al., 2007). In another study, tensile stress upregulated expression of OPG in synovial fibroblasts and decreased the RANKL/OPG ratio (Nazet et al., 2022).

### 6.2 TMJ disc

The TMJ disc is an avascular and noninnervated tissue composed of fibrocartilage with viscoelastic consistency and possesses transitional characteristics between those of fibrous tissue and cartilage. The disc is an essential element in the normal TMJ that has the following functions: i) provides a smooth interface between the condyle and the mandibular fossa; ii) provides load-bearing and support forces (e.g., compression, tension and shear forces); and iii) lubricates the surrounding surfaces for different ranges of motion (Vapniarsky et al., 2018; Trindade et al., 2021).

The normal disc ECM consists of collagen fibers (mainly type I collagen and type II collagen mostly found in the intermediate zone), glycosaminoglycan (GAG) and proteoglycans and elastic fibers. A summary of the influence of mechanical stresses on the ECM is as follows.

The magnitude of mechanical forces affects the responses of collagen fibers. The typical wavelike structure of the collagen fibrils on the disc was maintained in response to modest tension and compression. However, excessive mechanical activity can also cause damage to local collagen fibrils (Kang et al., 2006). In a UAC model, type I collagen and type II collagen are reduced (Zhang HY. et al., 2019). Severe compressive force reduces the number of collagen fibers in articular discs (Magara et al., 2012). Importantly, the length of exposure to mechanical force influences the response. Changes in the collagen network from a wavelike structure to distortion are reversible in short-term stimulation indentation, but severe, irreversible breakdown and deformation of the collagen-proteoglycan network occur in specimens that have been compressed for a long time (Kang et al., 2006).

Proteoglycans can be classified into 2 major families: modular proteoglycans (also called hyalectans) and small leucine-rich proteoglycans (SLRPs) (Iozzo and Murdoch, 1996). A previous study revealed that expression of versican (a modular proteoglycan), as well as biglycan, chondroadherin and decorin (a small leucine-rich proteoglycan), increased in response to stimulation by mechanical force (Nakao et al., 2015). Versican plays a central role in the initiation and increase in inflammatory processes (Zhang et al., 2012), wound healing and angiogenesis (Toriya et al., 2006; Choocheep et al., 2010; Michaelis et al., 2018). Chondroadherin is thought to contribute to communication between TMJ cells and matrices and to modulation of cellular activity and matrix assembly (Nakao et al., 2015). Decorin is predominant in the peripheral region under tensile force (Kuwabara et al., 2002), whereas biglycan is more prominent in the posterior band of the TMJ disc and is likely subjected to compressive force during biting (Kuwabara et al., 2002). The elevated proteoglycan expression of decorin and biglycan is interpreted as an increase in the magnitude of compressive forces in the TMJ (Mao et al., 1998). However, in another study, aggrecan, decorin and fibromodulin were downregulated in the UAC model group (Zhang HY. et al., 2019). These seemingly paradoxical results might be due to differences in the magnitude of the mechanical force applied.

Expression of GAG is modulated by mechanical forces. The content of GAG increased significantly in the bite plane group (Nakao et al., 2015). Specifically, stress stimulates expression of C6S compared with that in controls. As C6S is the major component of hyaline cartilage, compressive forces in the articular disk may stimulate the development of more cartilaginous-like properties with respect to GAG content (Carvalho et al., 1995).

Desmin is a type of intermediate filament that is a component of the cytoskeleton. Compressive force provoked a significant increase in desmin, suggesting that mature articular cells are capable of producing desmin in response to mechanical stress (Magara et al., 2012).

In addition to specific components, sustained mechanical loading may significantly alter the nutrient distribution inside the TMJ disc by impeding solute transport (Kuo et al., 2011; Wright et al., 2013), which subsequently causes restriction of nutrient supply and waste removal and may result in homeostasis disruption and initiate cell death (Wu et al., 2019b).

It is commonly accepted that TMJ disc cells consist mainly of fibroblasts and chondrocyte-like cells. Several scholars have concluded that there are three kinds of cells, namely, fibroblast-like cells (elongated and the greatest percentage of cells), fibrochondrocytes (rounded cells without a pericellular halo), and chondrocyte-like cells (rounded cells with a pericellular halo) (Leonardi et al., 2002). Dynamic tensile strain can downregulate the catabolic activity of fibrocartilage cells in an inflammatory environment by inhibiting expression of a variety of MMPs (including MMP-3, -7, -8, -9, -13, −16, −17, and −19) (Deschner et al., 2006). However, overload of mechanical force can induce upregulation of inflammatory cytokines and multiple subtypes of MMPs in TMJ disc cells, which is associated with TRPV4 activation and Ca^2+^ influx (Cui et al., 2023). Furthermore, aquaporin-1 (AQP-1) has been reported to mediate the effects of stress on TMJ disc cells (Loreto et al., 2012).

## 7 Discussions and future directions

### 7.1 The mechanotransduction signaling process involves three-tiered cascade

Condylar chondrocytes are mechanosensitive. Dependent on integrins, the cytoskeleton, ion channels and primary cilia, condylar chondrocytes are capable of converting mechanical stimuli into biochemical signals, thereby triggering subsequent biological responses. This process of mechanotransduction signaling involves mechanosensors, mechanotransducers, and mechanoimpactors (Wang et al., 2023). Mechanosensors are the elements that directly sense and transduce the mechanical signals. In fact, the four mechanosensitive elements that we have discussed at the third section, including integrins, the cytoskeleton, ion channels and primary cilia, belong to mechanosensors. Mechanotransducers refer to molecules that activated by upstream signaling pathways and modulate specific downstream molecules, which plays a bridging role in connecting mechanosensors and mechanoimpactors. Most of the critical signaling molecules that we discussed in this review belong to the mechanotransducers. Mechanoimpactors represent the final effector of mechanotransduction signaling, resulting in biological changes of chondrocytes and ECM. In this review, we classified the critical signaling molecules according to various mechanoimpactors. Notably, the abovementioned critical signaling molecules can also be activated by other factors not limited to mechanical stimulation. At present, molecules that only respond to mechanical stimulation have not been found, which deserve further exploration.

### 7.2 More accurate modeling methods are in urgent need

Different magnitudes of mechanical stimulation produce various effects on TMJ tissue, which are regulated by critical signaling molecules. Moderate mechanical stimulation is essential for maintaining the homeostasis whereas abnormal mechanical stimulation disturbs the balance. Evaluating the variation in critical signaling molecules is beneficial for determining whether mechanical loading on the TMJ is moderate, which holds promise for realizing early diagnosis and prevention of TMJ degenerative disease. However, the actual mechanical stress environment in the TMJ is very complicated and cannot be fully simulated by existing mechanical loading models (Table 1). In the future, modeling methods that closely approximate the real stress environment are urgently needed to determine the variation in critical signaling molecules more accurately.

**TABLE 1:** Mechanical loading models in this review and their effects.

Subjects	Species	Age/Passage number	Mechanical loading methods	Time	Effects	Ref.
Chondrocytes	Rat	3	Hydrostatic compressive forces at 50–250 kPa	2 h	Reduce apoptosis and enhance viability	Ma et al. (2016)
Chondrocytes	Rat	4–10	Hydrostatic pressure at 50–200 kPa	1 h	Promote catabolism and anabolism	Zhou et al. (2020b)
Chondrocytes	Rat	—	Hydrostatic pressure at 0.3 MPa	24 h	Promote apoptosis	Xu et al. (2017)
Chondrocytes	Rat	2	Cyclic compressive forces at 1,000–4,000 μstrain, 0.5 Hz	2 h	Promote catabolism	Li et al. (2022)
Chondrocytes	Rat	2	Negative pressure at 10 kPa	4 h	Promote chondrocyte proliferation	Liu et al. (2021b)
Chondrocytes	Rabbit	3–4	Continuous pressure at 90 kPa	1 h	Promote anabolism and inhibit inflammation	Chen et al. (2007)
Chondrocytes	Mouse	≤6	Uniaxial compression at 2.5 N	2 h	Promote catabolism	Reed et al. (2022)
Synovial fibroblasts	Rat	3–7	Intermittent hydrostatic pressure at 100 kPa	4 h/d for 2 d	Promote boundary lubrication (upregulation of PRG4)	Xu et al. (2012)
Synovial cells	Human	5–7	Cyclic compressive forces at 20–40 kPa, 0.5 Hz	1 h	Promote catabolism	Akamine et al. (2012)
Synovial cells	Human	6	Cyclic compressive forces at 20 kPa, 0.5 Hz	1 h/d for 5 d	Promote catabolism	Muroi et al. (2007)
Synovial cells	Rat	—	Compressive force at 2.0 g/cm^2^	12 h	Promote osteoclast formation	Ichimiya et al. (2007)
Synovial fibroblasts	Rat	—	Hydrostatic pressures at 30–120 kPa	12 h	Promote inflammation	Wu et al. (2013)
Disc cells	Rat	2–3	Continuous compressive force at 0.5–1.5 g/cm^2^	24 h	Promote catabolism and inflammation	Cui et al. (2023)
SW1353	Human	—	Static pressure at 150 kPa or 200 kPa	3 h	Promote catabolism	Fan et al. (2020)
Chondrocytes	Rat	—	CTS at 20% elongation, 0.1 Hz	6–12 h	Promote apoptosis	Xu et al. (2019)
Chondrocytes	Rat	3	CTS at 20% elongation, 0.1 Hz	2–12 h	Inhibit anabolism	Zhang et al. (2022a)
Chondrocytes	Pig	—	CTS at 7% elongation, 0.5 Hz	12–48 h	Promote boundary lubrication (upregulation of SZP)	(Kamiya et al., 2010)
		—	CTS at 21% elongation, 0.5 Hz		Inhibit synthesis of SZP	
Chondrocytes	Mouse	1	CTS at 6% elongation, 0.5 Hz	1–6 h	Promote proliferation	Liu et al. (2019a)
Cartilage	Rat	6 w	Mandibular propulsive appliance	2 w	Promote anabolism	(Sun et al., 2017)
Chondrocytes		2	CTS at 16% elongation, 1 Hz	3–12 h		
Chondrocytes	Pig	—	IL-1β + CTS at 6% elongation, 0.5 Hz	8 h	Inhibit inflammation	Tabeian et al. (2019)
Chondrocytes	Rabbit	—	IL-1β + CTS at 3%–9% elongation, 0.05 Hz	24 h	Inhibit inflammation	Agarwal et al. (2001)
Chondrocytes	Rat	3	CTS at 20% elongation, 0.5 Hz	24 h	Promote apoptosis	Huang et al. (2017)
Chondrocytes	Rat	—	CTS at 1,000–3,000 μstrain, 0.5 Hz	2 h	Promote catabolism	Li et al. (2017)
Synovial fibroblasts	Mouse	6–7	CTS at 15% elongation, 0.5 Hz	16 h and a break of 8 h in-between	Promote inflammation	Nazet et al. (2022)
ATDC5	Mouse	—	FFSS at 24 dyne/cm^2^	1–2 h	Promote autophagy	Yang et al. (2020b)
				1–4 h	Promote apoptosis	
Chondrocytes	Rat	—	FFSS at 12 or 24 dyne/cm^2^	1 h/w for 3 w	Promote abnormal adipogenesis	Wang et al. (2022b)
ATDC5	Mouse	—	FFSS at 12 or 24 dyne/cm^2^	1 h	Promote terminal differentiation and inhibit anabolism	Liu et al. (2018b)
Chondrocytes	Rat	—	FFSS at 16 dyne/cm^2^	1 h	Promote terminal differentiation and inhibit anabolism	Zhang et al. (2019a)
Superficial chondrocytes	Rat	—	FFSS at 16 dyne/cm^2^	2 h	Promote proliferation	Zhou et al. (2023)
Chondrocytes	Rat	—	FFSS at 16 dyne/cm^2^	1–4 h	Promote apoptosis	Ren et al. (2019)
Chondrocytes	Rat	—	FFSS at 8 or 16 dyne/cm^2^	1 h	Promote pathological calcification	Liu et al. (2022)
Cartilage	Rabbit	Adult	Anterior disc displacement	2 w	Promote degeneration	Zhou et al. (2020b)
Cartilage and subchondral bone	Mouse	3 w	Incisor trimming with soft diet	2–6 w	Promote degeneration	Chen et al. (2009)
Cartilage	Rat	2 w	Hard diet	6–48 h	Promote growth	Papachristou et al. (2006)
Cartilage	Mouse	8 w	Botulinum toxin A injection into masseters	4 w	Promote degeneration	(Hou et al., 2023)
			Hyperactivity biting			
Cartilage and subchondral bone	Mouse	6 w	Botulinum neurotoxin into masseters	4 w	Promote degeneration	(Dutra et al., 2018)
			Botulinum neurotoxin into masseters + forced mouth open	4 w + 1 h/d for 5 d	Rescue degeneration	
Cartilage	Rat	9 w	Steady mouth-opening at 2 N	2 h/d for 5 d	Promote degeneration	Ge et al. (2017)
Cartilage	Rat	5 w	Mandibular lateral shift	2–4 w	Promote boundary lubrication (upregulation of PRG4)	Yang et al. (2020a)
Cartilage	Rat	5 w	Mandibular propulsive appliance	7–21 d	Promote boundary lubrication (upregulation of lubricin)	Chen et al. (2019a)
Cartilage	Rat	5 w	Mandibular propulsive appliance	3–60 d	Promote growth	Ng et al. (2006)
Cartilage	Rabbit	8 w	Mandibular propulsive appliance	3 d-12 w	Promote endochondral ossification	Jing et al. (2013)
Cartilage	Rat	3 w	Mandibular propulsive appliance	5–15 d	Promote chondrocyte proliferation	Hajjar et al. (2003)
Cartilage	Rat	5 w	Mandibular propulsive appliance	1–17 d	Promote anabolism	Rabie et al. (2003a)
Cartilage and subchondral bone	Rat	5 w	Overloaded functional orthopedic force	2–8 W	Promote degeneration	He et al. (2022b)
Cartilage	Mouse	6 w	BAE	7–11 w	Promote proliferation	Liu et al. (2019a)
Cartilage	Mouse	6 w	UAC	7–15 w	Promote degeneration	Zhou et al. (2020a)
			UAC + BAE	7–15 w + 4w	Rescue degeneration	
Cartilage	Rat	3 w	UAC	8 w	Promote degeneration	Zhou et al. (2022)
Cartilage	Rat	6 w	UAC	2–20 w	Promote degeneration	Yang et al. (2020b)
Cartilage	Mouse	6 w	UAC	6–8 w	Promote degeneration	Qi et al. (2022a)
Cartilage	Mouse	6 w	UAC	1–11 w	Promote degeneration	Liu et al. (2020)
Subchondral bone	Rat	8 w	UAC	4–8 w	Promote degeneration	Yang et al. (2022)
Subchondral bone	Mouse	6 w	UAC	3 w	Promote degeneration	Sun et al. (2020a)
Subchondral bone	Rat	6 w	UAC	4 w	Promote degeneration	Yang et al. (2015)
Disc	Rat	6 w	UAC	4–20 w	Promote degeneration	Zhang et al. (2019b)
Disc	Rat	7 w	UAC	3 d	Promote degeneration and inflammation	Cui et al. (2023)
Cartilage	Rat	8 w	Molars movement	4–12 w	Promote degeneration	Zhang et al. (2013)
Cartilage	Rat	8 w	Molars movement	24 w	Promote degeneration and regeneration	Kuang et al. (2013)
Cartilage	Rat	8 w	A ligation silk knot (0.25 mm diameter) on the first molar of maxillary	2–8 w	Promote degeneration	Zhang et al. (2022b)
Cartilage	Mouse	8 w	A wire (0.012 diameter, 2.5 mm long) on molars of maxillary	2–8 w	Promote degeneration	Matias et al. (2016)
Cartilage	Rat	8 w	Occlusal elevation at 2 mm	2–8 w	Promote degeneration	Zhuang et al. (2023)
Disc	Rat	7 w	Occlusal elevation at 2 mm	1–4 w	Promote adaptive responses	Nakao et al. (2015)
Cartilage	Rat	9 w	Occlusal elevation at 1.5 mm for the first maxillary molar and 1 mm for the third molar	3–28 d	Promote degeneration	Long et al. (2019)
Cartilage	Rat	7 w	Compressive mechanical force at 80 g	4–7 d	Promote degeneration and apoptosis	Zhu et al. (2016)
Cartilage	Rat	8 w	Compressive mechanical force at 40 g	2 w	Promote degeneration	Li et al. (2013)
Cartilage	Mouse	8 w	Compressive mechanical force	1 w	Promote degeneration	Du et al. (2020b)
Disc	Rat	8 w	Compressive mechanical force at 50 g	5 d	Promote degeneration	Magara et al. (2012)

### 7.3 Changes of mechanical stimulation may influence the final output of critical signaling

We notice that several signaling molecules activated by moderate mechanical loading, such as PRG4, HMGB2 and PTHrP, can also exert compensatory effects under abnormal mechanical stimulation, indicating that they play the same homeostasis-promoting role under different mechanical stress conditions (Yang W. et al., 2020; Zhou Y. et al., 2020; Zhang et al., 2022c). Then, whether all the signaling molecules can maintain the final effect unchanged if the mechanical stress condition is altered? In fact, some signaling molecules play different roles when stimulated by different mechanical loadings. For example, TRPV4 is able to promote anabolism under moderate mechanical stress but induce apoptosis when the stress is excessive (O’Conor et al., 2014; Xu et al., 2019). Moderate expression of VEGF is beneficial for the endochondral ossification, but overexpression of VEGF induced by abnormal mechanical stimulation can result in catabolism and subchondral bone loss (Jiao et al., 2011; Jiang et al., 2017). In conclusion, we speculate that there will always be some mechanosensitive signaling pathways changed when the mechanical stress condition is changed, thereby interfering with the downstream pathways of critical singaling moleclues, which could influence the final output. Due to the complicated molecular network, the detailed mechanisms remain to be elucidated.

### 7.4 Critical signaling molecules have potential therapeutic implications

Molecules with compensatory effects attempt to impede TMJOA progression. They not only facilitate proliferation, differentiation, clearance, anabolism and matrix crosslinking in condylar cartilage but also promote subchondral bone formation; additionally, they play inhibitory roles in terminal differentiation, apoptosis and aberrant lipid metabolism. Molecules with decompensatory effects accelerate TMJ degeneration. They function in promoting cell death, catabolism, terminal differentiation, pathological calcification and abnormal subchondral bone remodeling. Based on the above studies, we discover that if molecules with compensatory effects occupy a dominant position under abnormal mechanical stimulation, the condyle homeostasis can be effectively maintained, thereby delaying even reversing the degeneration. In contrast, if molecules with decompensatory effects are more predominant, the homeostasis can be disrupted, accelerating the degeneration process. Therefore, the treatment for TMJOA based on signaling molecules could be approximately categorized into two directions: enhancing compensatory molecules-mediated signaling or inhibiting decompensatory molecules-mediated signaling. For example, intra-articular injection of adenovirus (Ad-Rap2a-GFP) overexpressed compensatory RAP2A molecules and then alleviated TMJOA lesions of UAC-treated mice (Qi et al., 2022a). Besides, specific inhibition of decompensatory BRD4 molecules by JQ1 attenuated the degenerative changes in rat condyle that induced by compressive mechanical force (Huang Z. et al., 2021). However, TMJOA is a highly heterogeneous disease involved in a complicated network of signaling molecules, thus focusing on a single molecular target may not achieve desirable therapeutic effect, suggesting that combination of multiple targeted molecules is a future direction for TMJOA treatment. In addition, the TMJ is covered by a synovial membrane and is composed of the mandibular condyle, glenoid fossa and disc. All these components, not limited to the condyle, can be affected by mechanical loading. Whether the signaling crosstalk among them will affect the treatment outcome need to be fully considered. Moreover, based on the fact that moderate mechanical stimulation is beneficial for maintaining the TMJ homeostasis, only paying attention to therapeutic targets is not sufficient. At the same time, it is necessary to improve the adverse stress environment in the TMJ cavity through occlusal splints, occlusal adjustment and orthodontic or orthognathic treatment.

## Figures and Tables

**Table:** 

**TMJ**	temporomandibular joint
**TMJOA**	temporomandibular joint osteoarthritis
**ECM**	extracellular matrix
**FAK**	focal adhesion kinase
**ILK**	integrin-linked kinase
**IP3**	inositol triphosphate
**2APB**	aminoethoxydiphenyl borate
**ROR**	ryanodine receptorchannels
**Rya**	ryanodine
**CaMKII**	calmodulin-dependent protein kinase II
**MAPK**	mitogen-activated protein kinase
**ER**	endoplasmic reticulum
**HCF**	hydrostatic compressive force
**ERK**	extracellular signal–regulated kinase
**PI3K**	phosphatidylinositol-3-kinase
**MLC-II**	myosin light chain II
**TRPV4**	transient receptor potential cation channel subfamily V member 4
**CTS**	cyclic tensile strain
**UAC**	unilateral anterior crossbite
**mTOR**	mammalian Target of rapamycin
**IFT**	intraflagellar transport
**PGE2**	prostaglandin E2
**PRG4**	proteoglycan 4
**TNFα**	tumor necrosis factor α
**SZP**	superficial zone protein
**IL**	interleukin
**HMGB2**	high-mobility group box 2
**IGF**	insulin-like growth factor
**IGFR1**	insulin-like growth factor 1 receptor
**IGFBP**	insulin-like growth factor-binding protein
**RKIP**	Raf kinase inhibitor protein
**MMP**	matrix metalloproteinase
**NO**	nitric oxide
**PTHrP**	parathyroid hormone-related protein
**PTH1R**	parathyroid hormone receptor 1
**SOX9**	sex-determining region Y-box 9
**YAP**	Yes-associated protein
**ADAMTS5**	a disintegrin and metalloproteinase with thrombospondin motif 5
**RUNX2**	runt-related transcription factor 2
**AP-1**	activator protein-1
**JNK**	c-Jun N-terminal kinase
**BAE**	bilateral anterior elevation
**PCNA**	proliferating cell nuclear antigen
**MSC**	mesenchymal stem cell
**VEGF**	vascular endothelial growth factor
**Ang**	angiopoietin
**LC3**	light chain 3
**MAP4K3**	mitogen-activated protein kinase kinase 3
**CD163**	cluster of differentiation 163
**NG2**	neuron-glial antigen 2
**CSPG4**	chondroitin sulfate proteoglycan 4
**CCL2**	C-C motif chemokine ligand 2
**MCP1**	monocyte chemoattractant protein 1
**PDGFrβ**	platelet-derived growth factor receptor beta
**RAP2A**	Ras-related protein Rap-2a
**HIF**	hypoxia-inducible factor
**PCM**	pericellular matrix
**OPG**	osteoprotegerin
**RANKL**	receptor activator of nuclear factor kappa-B ligand
**GDF**	growth differentiation factor
**LOX**	lysyl oxidase
**CaSR**	calcium-sensing receptor
**APAF-1**	apoptotic protease activating factor-1
**Cyt C**	cytochrome C
**CHOP**	C/EBP homologous protein
**ERS**	endoplasmic reticulum stress
**ERN1**	endoplasmic reticulum to nucleus signaling 1
**ATF6**	activating transcription factor 6
**EIF2AK3**	eukaryotic translation initiation factor 2 alpha kinase 3
**MTORC1**	mechanistic target of rapamycin complex 1
**FFSS**	flow fluid shear stress
**GRP**	glucose regulated protein
**HP**	hydrostatic pressure
**SIRT1**	silent information regulator 1
**UTR**	untranslated region
**RIP1**	receptor interacting protein kinase 1
**MLKL**	mixed lineage kinase domain-like protein
**SDC4**	syndecan 4
**DAMP**	damage-associated molecular pattern
**GPX4**	glutathione peroxidase 4
**ACSL4**	acyl coenzyme A synthetase long chain family, member 4
**NLRP3**	NOD-like receptor protein 3
**GSDMD**	gasdermin D
**MIA**	miodoacetate
**SASP**	senescent-associated secretory phenotype
**m6A**	N6-methyladenosine
**METTL3**	methyltransferase-like 3
**TLR4**	toll-like receptor 4
**TGF**	transforming growth factor
**HtrA1**	high temperature requirement A1 serine protease
**TCF/LEF**	T-cell factor/lymphoid enhancer factor
**PKA**	protein kinase A
**NF-κB**	nuclear factor kappa B
**SDF**	stromal cell-derived factor
**CXCR4**	CXC receptor 4
**BRD4**	bromodomain containing 4
**TREM1**	triggering receptor expressed on myeloid cells 1
**EP4**	prostaglandin E receptor 4
**RAGE**	receptor for advanced glycation end products
**Ihh**	Indian hedgehog
**ALP**	alkaline phosphatase
**Ptch1**	Patched 1
**Smo**	Smoothened
**MV**	matrix vesicle
**EV**	extracellular vesicle
**HDAC6**	histone deacetylase 6
**TNAP**	tissue-nonspecific alkaline phosphatase
**MGP**	matrix Gla protein
**Ror2**	receptor tyrosine kinase-like orphan receptor 2
**CXCL12**	chemokine C-X-C motif ligand 12
**NFAT**	nuclear factor of activated T cells
**Adrb2**	β2-adrenergic receptor
**Sema4D**	Semaphorin 4D
**CCR**	chemokine receptor
**SP1**	specific protein 1
**SF**	synovial fibroblast
**Has1**	hyaluronic acid synthase 1
**Cemip2**	cell-migration-inducing hyaluronidase 2
**Fn1**	Fibronectin 1
**HA**	hyaluronic acid
**Icam-1**	intercellular adhesion molecule-1
**Ptgs2**	prostaglandin-endoperoxide synthase 2
**EDP**	elastin derived peptide
**COX-2**	cyclooxygenase-2
**iNOS**	inducible nitric oxide synthase
**GAG**	glycosaminoglycan
**SLRP**	small leucine-rich proteoglycans
**AQP-1**	aquaporin-1
